# ZDHHC18 promotes renal fibrosis development by regulating HRAS palmitoylation

**DOI:** 10.1172/JCI180242

**Published:** 2025-02-04

**Authors:** Di Lu, Gulibositan Aji, Guanyu Li, Yue Li, Wenlin Fang, Shuai Zhang, Ruiqi Yu, Sheng Jiang, Xia Gao, Yuhang Jiang, Qi Wang

**Affiliations:** 1 Nephrology Department, Guangzhou Women and Children’s Medical Center, Guangzhou Medical University, Guangzhou, China.; 2 Department of Endocrinology, The First Affiliated Hospital of Xinjiang Medical University, State Key Laboratory of Pathogenesis, Prevention and Treatment of High Incidence Diseases in Central Asia, Urumqi, China.; 3 Department of Pediatrics, Jiangxi Children’s Medical Center, Nanchang, China.; 4 State Key Laboratory of Genetic Engineering, School of Life Sciences and Zhongshan Hospital, Fudan University, Shanghai, China.; 5 The Key Laboratory of Experimental Teratology of the Ministry of Education and Department of Histology and Embryology, School of Basic Medical Sciences, Cheeloo College of Medicine, Shandong University, Jinan, China.; 6 Department of Orthopedics, The Eighth Affiliated Hospital of Sun Yat-sen University, Shenzhen, China.; 7 Qingyuan People’s Hospital, The Sixth Affiliated Hospital of Guangzhou Medical University, Qingyuan, China.

**Keywords:** Nephrology, Therapeutics, Fibrosis

## Abstract

Fibrosis is the final common pathway leading to end-stage chronic kidney disease (CKD). However, the function of protein palmitoylation in renal fibrosis and the underlying mechanisms remain unclear. In this study, we observed that expression of the palmitoyltransferase ZDHHC18 was significantly elevated in unilateral ureteral obstruction (UUO) and folic acid–induced (FA-induced) renal fibrosis mouse models and was significantly upregulated in fibrotic kidneys of patients with CKD. Functionally, tubule-specific deletion of ZDHHC18 attenuated tubular epithelial cells’ partial epithelial-mesenchymal transition (EMT) and then reduced the production of profibrotic cytokines and alleviated tubulointerstitial fibrosis. In contrast, ZDHHC18 overexpression exacerbated progressive renal fibrosis. Mechanistically, ZDHHC18 catalyzed the palmitoylation of HRAS, which was pivotal for its translocation to the plasma membrane and subsequent activation. HRAS palmitoylation promoted downstream phosphorylation of MEK/ERK and further activated Ras-responsive element–binding protein 1 (RREB1), enhancing SMAD binding to the *Snai1* *cis*-regulatory regions. Taken together, our findings suggest that ZDHHC18 plays a crucial role in renal fibrogenesis and represents a potential therapeutic target for combating kidney fibrosis.

## Abstract 2

Our findings suggest that ZDHHC18 plays a pivotal role in renal fibrogenesis and presents a potential therapeutic target for combating kidney fibrosis.

## Graphical abstract

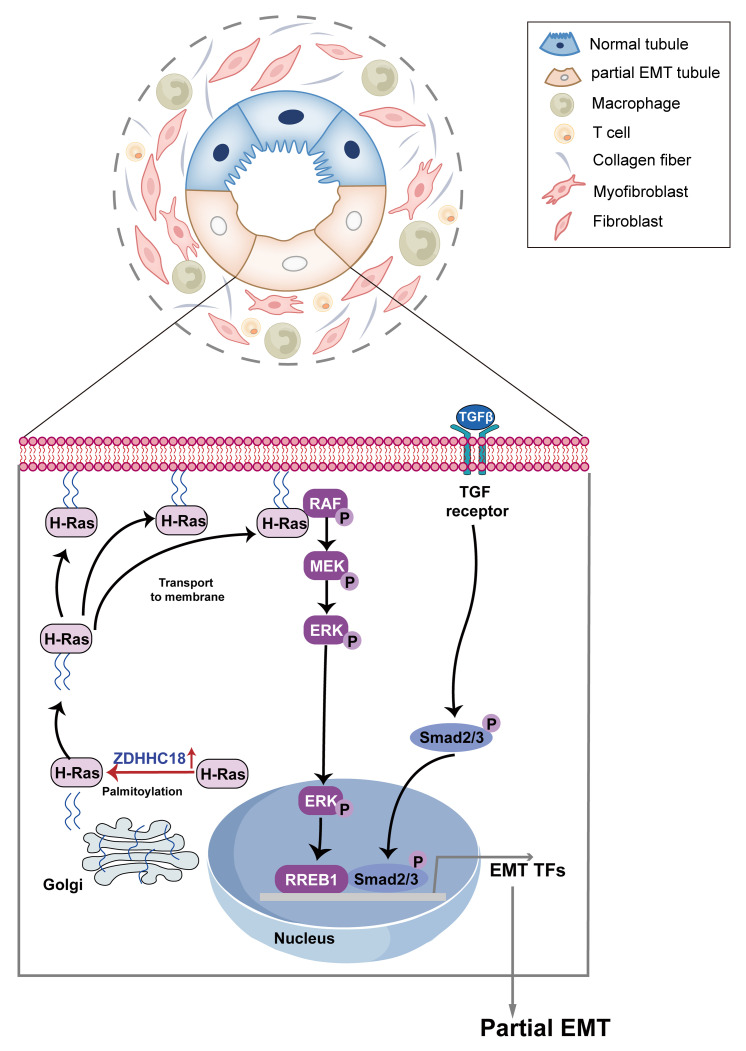

## Introduction

Almost all forms of chronic kidney disease eventually progress to renal fibrosis (1, 2). Tubular epithelial cells (TECs) are the main component of the kidney. When kidney injury occurs, injured TECs undergo partial epithelial-mesenchymal transition (EMT) while still residing within the basement membrane of the tubules. They are characterized by their acquisition of mesenchymal features and coexpression of both epithelial and mesenchymal cell markers. TECs undergoing partial EMT release including proinflammatory and profibrotic factors into the renal interstitium, thereby remodeling the microenvironment to promote inflammation and fibrosis (3–5). Therefore, identifying key molecules involved in the partial EMT process in TECs may lead to the development of therapeutic approaches for preventing renal fibrosis.

Protein S-palmitoylation is a common posttranslational modification that increases the hydrophobicity of proteins and plays an important role in regulating protein transport, location, and functional activation (6, 7). S-palmitoylation links palmitate with specific cysteine residue (Cys) side chains of proteins through unstable thioester bonds. This modification is reversible (8, 9). Protein palmitoylation is catalyzed by a series of enzymes called zinc finger DHHC (ZDHHC) palmitoyltransferases, which contain the signature Asp-His-His-Cys (DHHC) motif (10). ZDHHC protein family members are involved in various physiological and pathological processes. ZDHHC13 has been reported to catalyze palmitoylation of the GPCR MC1R to inhibit the development of melanoma (11). ZDHHC8-KO mice exhibit prepulse inhibition defects, leading to behavioral abnormalities (12, 13). Previous studies have reported that polycystin 1 (PKD1) palmitoylation increases the protein level of PKD1 and promotes the occurrence of polycystic kidney disease (14). β-Catenin palmitoylation leads to protein degradation and inhibits the occurrence of renal fibrosis (15). However, as many as 23 ZDHHC enzymes can catalyze the s-palmitoylation of proteins. The role and function of the ZDHHC enzyme in renal fibrosis are not fully understood.

*RAS* is a well-known oncogene that regulates cell survival, growth, and differentiation (16, 17). *RAS* has 3 isoforms, *HRAS*, *NRAS* and *KRAS*. The sequences of these *RAS* isoforms share a high degree of sequence homology, but they have different biological effects (18). HRAS and the downstream MEK/ERK pathways are activated by unilateral ureteral obstruction (UUO) (19–21), and KO of HRAS in mice reduces UUO-induced renal fibrosis (22). The activation of RAS signaling depends on the subcellular localization of GTPase (23). HRAS is palmitoylated by ZDHHC9 in the Golgi apparatus. Palmitoylation greatly improves the affinity of HRAS for the plasma membrane (PM). HRAS is recruited to the PM and further activated by receptor-Grb2-SOS complexes. Activated HRAS proteins recruit RAF to the PM, where it becomes active and initiates the MEK/ERK signaling cascade (24). However, ZDHHC9 expression is significantly downregulated during renal fibrosis (15). So, how HRAS is activated during renal fibrosis and whether other ZDHHC family palmitoyltransferases modify its palmitoylation remain unknown.

Here, we found that the expression of ZDHHC18 was markedly upregulated during renal fibrosis. Knocking out ZDHHC18 in renal TECs inhibited the expression of partial EMT–related genes and alleviates renal fibrosis phenotypes in vivo. Mechanistically, ZDHHC18 catalyzes HRAS palmitoylation, facilitating its localization to the plasma membrane. HRAS palmitoylation activated Ras-responsive element–binding protein 1 (RREB1), promoting SMAD binding to the *Snai1* and *Has2* *cis*-regulatory regions. Collectively, our results demonstrate that ZDHHC18 may be an attractive therapeutic target for treating kidney fibrosis.

## Results

### ZDHHC18 is upregulated in fibrotic kidneys of patients with chronic kidney disease.

**Figure 1:**
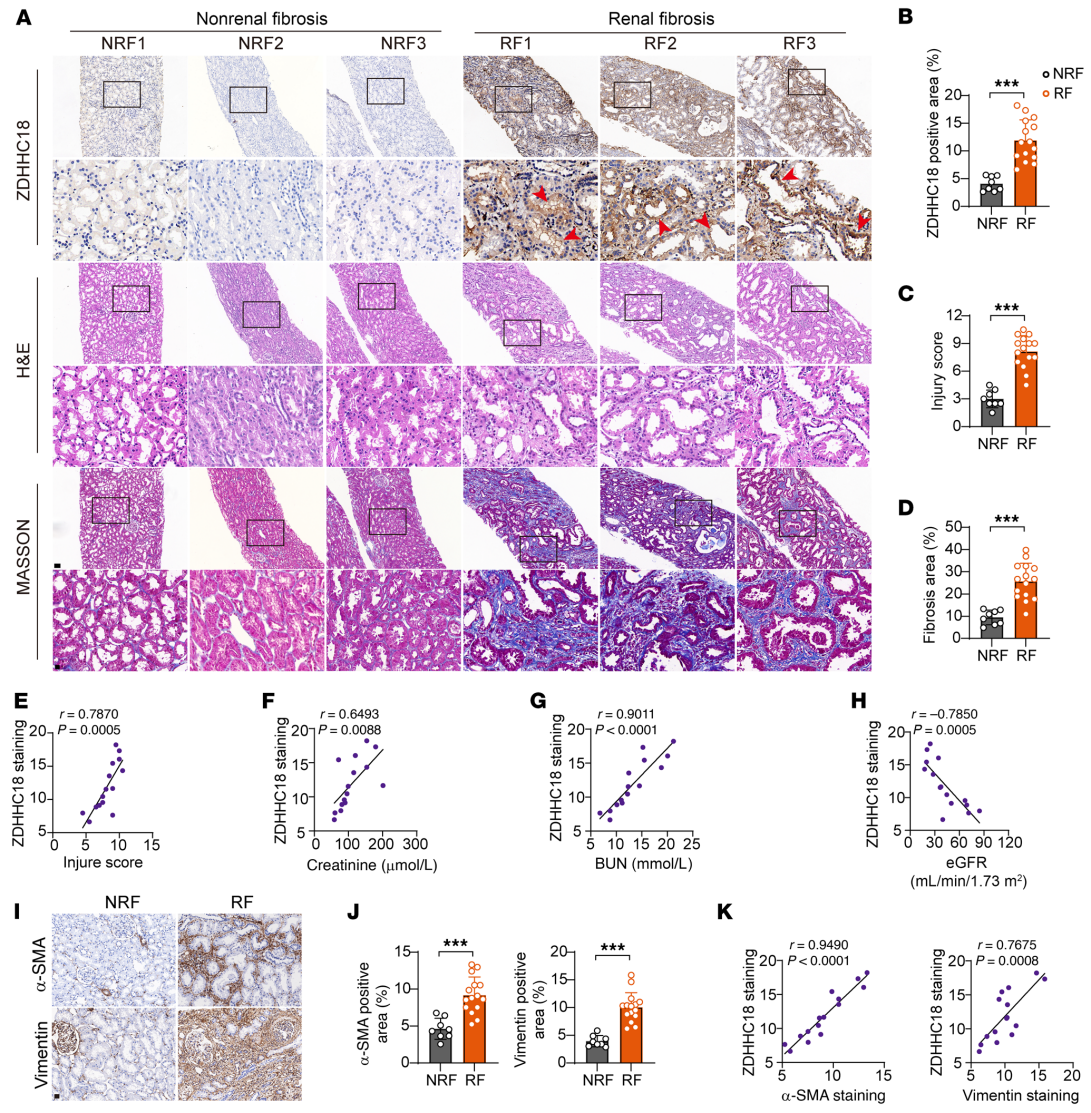
The expression of ZDHHC18 is markedly increased in the kidneys of patients with CKD. (**A**) Photomicrographs of ZDHHC18, H&E, and Masson staining in kidney sections from patients with nonrenal fibrosis (NRF) or renal fibrosis (RF). Red arrows indicate damaged tubules. Scale bars: 100 μm (enlarged insets: 20 μm). (**B**–**D**) Quantification of ZDHHC18 expression levels (**B**), kidney injury score (**C**), and fibrosis area (**D**) (*n* = 8 NRF, *n* = 15 RF). (**E**–**H**) Pearson’s correlation analysis showing the relationship between ZDHHC18 staining intensity and kidney injury score (**E**), sCr levels (**F**), BUN levels (**G**), and eGFR (**H**) in patients with RF (*n* = 15). (**I**) Photomicrographs of α-SMA and vimentin staining in kidney sections from the NRF and RF groups. Scale bar: 20 μm. (**J**) Quantitative analysis of α-SMA and vimentin staining in NRF (*n* = 8) and RF (*n* = 15) groups. (**K**) Pearson’s correlation analysis between ZDHHC18 levels and α-SMA and vimentin staining in the RF group (*n* = 15). Data are presented as the mean ± SD. ***P < 0.001, by 2-tailed Student’s *t* test (**B**–**D** and **J**).

We detected ZDHHC18 expression in microdissected kidney samples from patients with chronic kidney disease (CKD). The basic characteristics of the patients are summarized in Supplemental Table 1 (supplemental material available online with this article; https://doi.org/10.1172/JCI180242DS1). CKD samples showed significant interstitial fibrosis and tubular injury, as evidenced by Masson and H&E staining, compared with nonfibrotic kidney tissues (Figure 1, A, C, and D). IHC showed minimal ZDHHC18 expression in nonrenal fibrosis tissue but intense staining in fibrotic kidneys, predominantly in dilated proximal tubules lined by flat, thin epithelium lacking brush borders (Figure 1, A and B). Furthermore, ZDHHC18 levels showed significant positive correlations with the tubular injury score (Figure 1E), serum creatinine (sCr) levels (Figure 1F), and blood urea nitrogen (BUN) levels (Figure 1G). However, ZDHHC18 levels were negatively correlated with the estimated glomerular filtration rate (eGFR) (Figure 1H). The expression of α smooth muscle actin (α-SMA) and vimentin was markedly elevated in the kidney interstitium of fibrotic kidneys (Figure 1I). Linear regression analysis revealed a strong positive correlation between ZDHHC18 expression and the levels of both α-SMA and vimentin (Figure 1, J and K), indicating that ZDHHC18 played a significant role in kidney fibrosis.

### Zdhhc18 expression is upregulated in fibrotic kidneys of mice.

**Figure 2:**
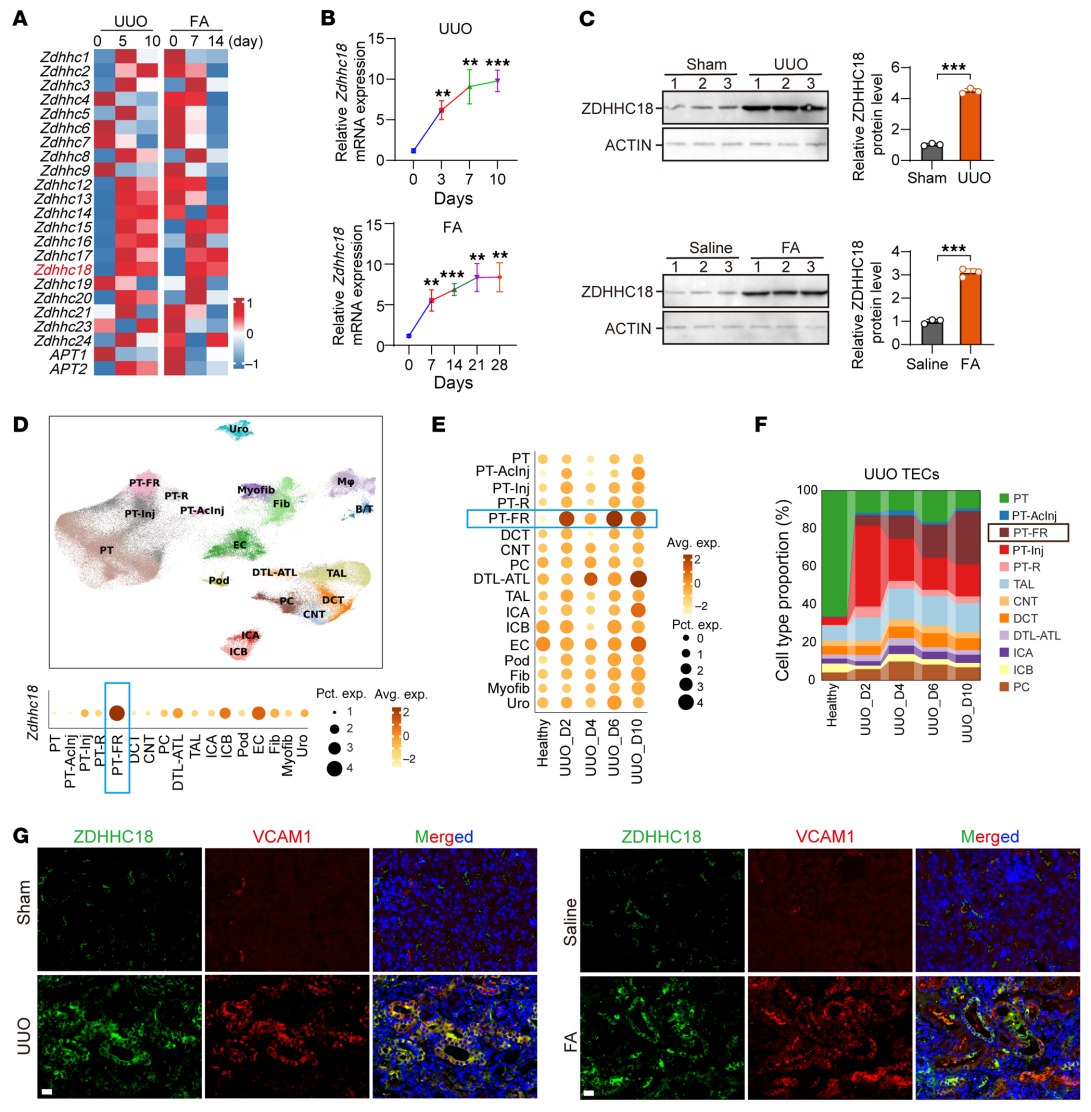
*Zdhhc18* is elevated in mouse fibrotic kidneys. (**A**) Heatmap of *ZDHHCs* and *APTs* gene expression in mouse kidneys after UUO (GSE125015) and FA injection (GSE65267). (**B**) *Zdhhc18* mRNA levels in mouse UUO kidneys (0, 3, 7, and 10 days) and at FA kidneys (0, 7, 14, 21, and 28 days) (*n* = 4). (**C**) WB analysis of ZDHHC18 expression in kidneys after 10 days of UUO and 28 days of FA, with ZDHHC18 levels quantified using ImageJ software (NIH) (*n* = 3). (**D**) Distribution and relative expression of *Zdhhc18* in different types of renal cells from mouse kidneys after UUO (GSE190887). (**E**) Relative expression of *Zdhhc18* on different days and cell subpopulations in UUO mouse kidneys. (**F**) Connected bar plots displaying the proportional abundance of subpopulations of TECs in different days of UUO. (**G**) Confocal microscopy images show staining for ZDHHC18 (green), VCAM1 (red), and DAPI (blue) in UUO (left) and FA (right) kidneys. Scale bars: 20 μm. Data are presented as the mean ± SD. **P < 0.01 and ***P < 0.001, by 2-tailed Student’s *t* test. PT, proximal tubule; PT-AcInj, acute injury PT; PT-Inj, injured PT; PT-R, repairing PT; DCT, distal convoluted tubule; CNT, connecting tubule; PC, principal cell of collecting duct; DTL, descending limb of loop of Henle (LoH); ATL, thin ascending limb of the LoH; TAL, thick ascending limb of the LoH; ICA, type A intercalated cell of the collecting duct; ICB, type B intercalated cell of the collecting duct; EC, endothelial cell; Pod, podocyte; Fib, fibroblast; Myofib, myofibroblast; Uro, urothelium; avg. exp., average expression; pct. exp., percentage of expression.

RNA-Seq data showed *Zdhhc18* upregulation in fibrotic kidneys of UUO mice or mice with folic acid–induced (FA-induced) renal fibrosis (Figure 2A). We confirmed these findings using UUO and FA mouse models (Supplemental Figure 1A). Quantitative real-time PCR (qRT-PCR) and Western blot (WB) analysis confirmed that the expression of ZDHHC18 was upregulated during renal fibrosis (Figure 2, B and C). Only a few ZDHHC family members (*Zdhhc14, Zdhhc15, Zdhhc17, Zdhhc18*, and *Zdhhc24*) were upregulated by both UUO and FA mice, and *Zdhhc18* showed the highest upregulation (Supplemental Figure 1B). *Apt1* and *Apt2*, thought to be responsible for depalmitoylation, were not upregulated during UUO- or FA-induced renal fibrosis (Figure 2A and Supplemental Figure 1C). Next, we examined the expression of fibrosis markers and their correlation with *Zdhhc18* expression. Our findings revealed a significant increase in the mRNA expression of *Col1a1*, *Col3a1, Fn1*, and *Acta2* in fibrotic kidneys compared with that in the control group (Supplemental Figure 2A). Among all *Zdhhc* family members, *Zdhhc18* exhibited the strongest positive correlation with these fibrosis markers (Supplemental Figure 2, B and C). Single-cell combinatorial indexing RNA-Seq (sci-RNA-Seq) analysis revealed that *Zdhhc18* had highest cumulative expression in the failed repair of the proximal tubule (PT-FR) subtype during UUO progression (Figure 2D) and displayed the most significant upregulation in PT-FR and descending limb–thin ascending limb of the loop of Henle (DTL-ATL) subtypes at day 10 after UUO compared with healthy kidneys (Figure 2E). Both PT-FR, a proximal tubule subtype, and DTL-ATL, part of the loop of Henle, belong to TECs. Notably, PT-FR became the largest TEC subpopulation in late-stage UUO (from day 6 to day 10) (Figure 2F). sci-RNA-Seq data showed that ZDHHC18 expression was also slightly upregulated in endothelial cells. Immunofluorescence experiments showed that ZDHHC18 was markedly increased in the VCAM1^+^ PT-FR cells of UUO and FA mice (Figure 2G), but no obvious changes were observed in CD31^+^ endothelial cells (Supplemental Figure 1D). These findings suggest that *Zdhhc18* abundance was predominantly upregulated in TECs from fibrotic mouse kidneys.

### ZDHHC18 enhances the TGF-β1–induced partial EMT in TECs.

To explore the role of ZDHHC18 upregulation in TECs, we next established an in vitro cell model by culturing human tubular epithelial HK-2 cells in the presence of TGF-β1. In response to TGF-β1 stimulation, ZDHHC18 expression was upregulated at both the mRNA (Supplemental Figure 3A) and protein levels (Supplemental Figure 3B). *ZDHHC18* knockdown in HK-2 cells (Supplemental Figure 3, C and D) resulted in upregulation of E-cadherin (*CDH1*), an epithelial cell marker, when treatment with TGF-β1 (Supplemental Figure 3E). The expression levels of TGF-β1–induced mesenchymal markers (*SNAI1*, *SNAI2*, and *VIM*) and fibrosis markers (*COL1A1*, *COL3A1*, *FN1*, and *ACTA2*) were significantly downregulated in response to *ZDHHC18* knockdown (Supplemental Figure 3, E and F). In contrast, overexpression of *ZDHHC18* (Supplemental Figure 3, G and H) increased the expression of TGF-β1–induced mesenchymal markers and fibrosis markers (Supplemental Figure 3, I and J).

Next, we generated mice with TEC-specific deletion of *Zdhhc18* using a conditional gene-targeting approach based on *Cre*/loxP recombination (Supplemental Figure 4A). Mice that were homozygous for the *Zdhhc18*-loxP–targeted allele (*Zdhhc18^fl/fl^*) were bred with TEC-specific *Cdh16* *Cre* lines, which was confirmed by tail genotyping (Supplemental Figure 4B). WB analysis confirmed the reduction of ZDHHC18 protein levels specifically in renal tubules of *Zdhhc18* conditional-KO (*Zdhhc18-*CKO) mice, with no detectable changes in glomeruli or endothelial cells (Supplemental Figure 4C). We also established an in vitro cell model of partial EMT by culturing primary TECs (PTECs) from *Zdhhc18-*CKO and WT mice. Following TGF-β1 treatment, PTECs from WT mice exhibited increased expression of mesenchymal markers (*Snai1*, *Snai2*, and *Vim*) concomitant with reduced expression of the epithelial marker *Cdh1*. Whereas *Zdhhc18-*CKO PTECs showed lower expression of TGF-β1–induced mesenchymal markers and higher expression of the epithelial marker *Cdh1*, these data for PTECs together with the data for HK-2 cells suggest that *Zdhhc18* promotes TGF-β1–induced partial EMT in vitro. In the process of renal fibrosis, TECs undergoing partial EMT contribute to fibroblast activation and inflammatory niche formation through TGF-β1 and proinflammatory cytokines secretion (25, 26). Our results indicate that *Zdhhc18* deficiency attenuated TGF-β1–induced expression of *Tgfb1*, a key cytokine for fibroblast activation (Supplemental Figure 4E). In addition, *Zdhhc18* KO suppressed the expression of proinflammatory cytokines and chemokines (*Il1b*, *Il6*, *Tnfa*, *Ccl2*, and *Ccl5*) (Supplemental Figure 4F).

### TEC-specific Zdhhc18 deletion inhibits renal fibrosis.

**Figure 3:**
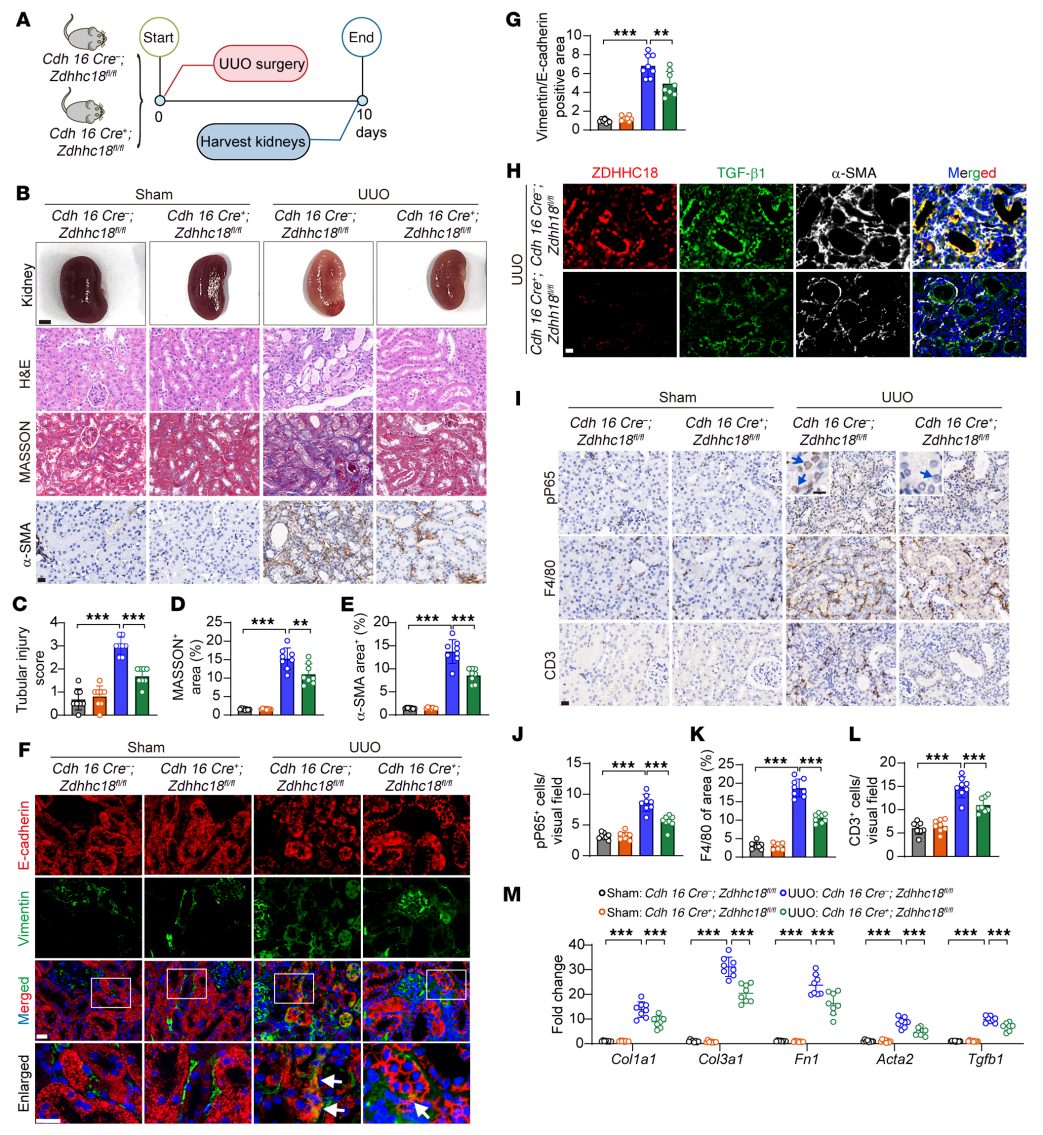
TEC-specific *Zdhhc18* deficiency inhibits renal fibrosis induced by UUO in mice. (**A**) Experimental design. Kidneys from WT and *Zdhhc18*-CKO mice were harvested after sham or UUO surgery for 10 days. (**B**) Gross appearance of the kidneys (scale bar: 2 mm) as well as images of H&E, Masson, and α-SMA staining of WT and *Zdhhc18*-CKO mouse kidneys after UUO. Scale bar: 20 μm. (**C**–**E**) Quantification of the tubular injury score (**C**), Masson staining of interstitial collagen (**D**), and α-SMA^+^ area (**E**) (*n* = 8). (**F**) Immunofluorescence images of staining. Square frames highlight digital enlargement of the tubule; white arrows indicate costaining for vimentin and E-cadherin. Scale bars: 20 μm. (**G**) Statistical analysis showing the percentage of vimentin- and E-cadherin–stained areas (*n* = 8). (**H**) Immunofluorescence images of ZDHHC18 (red), TGF-β1 (green), and α-SMA (white) expression in the kidneys of *Zdhhc18*-CKO mice after UUO. Scale bar: 20 μm. (**I**) p-P65, F4/80, and CD3 staining of kidneys from WT and *Zdhhc18*-CKO mice after UUO. Blue arrow indicates pP65^+^ cells in the renal tubules. Scale bars: 20 μm and 10 μm (enlarged insets). (**J**–**L**) Quantification of the proportion of pP65^+^ cells (**J**), F4/80^+^ area (**K**), and proportion of CD3^+^ cells (**L**) (*n* = 8). (**M**) mRNA levels of fibrotic markers in kidneys of *Zdhhc18* CKO and WT mice (*n* = 8). Data are presented as the mean ± SD. **P < 0.01 and ***P < 0.001, by 2-way ANOVA with Tukey’s multiple-comparison test (**C**–**E**, **G**, and **J**–**M**).

The *Zdhhc18*-CKO mice were born without any apparent abnormalities. At 2 months of age, there were no significant differences in terms of body weight (Supplemental Figure 5A), kidney weight (Supplemental Figure 5B), sCr levels (Supplemental Figure 5C), or BUN levels (Supplemental Figure 5D) between the *Zdhhc18-*CKO mice and *Zdhhc18^fl/fl^* littermates without *Cre* (WT). Furthermore, under normal conditions, we observed no apparent alterations in kidney structure (Figure 3B), indicating that specific deletion of *Zdhhc18* in TECs did not lead to phenotypic changes in mice.

In response to UUO (Figure 3A), *Zdhhc18* KO significantly improved kidney morphology and attenuated tubular injury, as shown by H&E staining (Figure 3, B and C). Compared with those in the sham control group, mice subjected to UUO displayed significant extracellular matrix (ECM) accumulation, but tubule-specific deletion of *Zdhhc18* decreased the extent of renal tubulointerstitial fibrosis, as demonstrated by Masson and Picrosirius red staining (Figure 3, B and D, and Supplemental Figure 5, E and F). Periodic acid–Schiff (PAS) staining revealed significant tubular dilatation and atrophy in the obstructed kidneys. However, these changes were much milder in *Zdhhc18*-CKO mice than in WT mice (Supplemental Figure 5E). Moreover, the interstitial accumulation of α-SMA^+^ myofibroblasts was upregulated by UUO, but the upregulation of α-SMA^+^ myofibroblasts was significantly reduced by *Zdhhc18* KO (Figure 3, B and E). Immunofluorescence analysis revealed that UUO induced partial EMT, as indicated by the presence of remaining TECs on the basement membrane and coexpression of the epithelial cell marker E-cadherin and the mesenchymal cell marker vimentin. However, this partial EMT progression was inhibited by *Zdhhc18* KO (Figure 3, F and G). Injured epithelial cells produce TGF-β1, which promotes the proliferation and activation of interstitial fibroblasts (25). Inhibition of partial EMT in TECs downregulates TGF-β1 expression and consequently attenuates fibroblast activation (26). Consistent with this, we found that *Zdhhc18* KO reduced TGF-β1 expression in renal tubules after UUO and decreased the number of interstitial α-SMA^+^ myofibroblasts (Figure 3H). We further found that *Zdhhc18* deficiency mitigated inflammatory reactions by decreasing the levels of proinflammatory mediators, such as *Il1b*, *Il6*, *Il18*, and *Tnfa* (Supplemental Figure 5G), and chemokines, such as *Ccl1*, *Ccl2*, *Ccl3*, *Ccl4*, and *Ccl5* (Supplemental Figure 5H), suppressed nuclear phosphorylated NF-κB (p-P65) levels in tubular cells and F4/80^+^ macrophages and CD3^+^ T cell infiltration (Figure 3, I–L). Attenuated inflammation and decreased fibroblast activation collectively resulted in mitigated fibrosis in the kidneys of *Zdhhc18*-KO mice. qRT-PCR further confirmed that the expression of the fibrosis markers *Col1a1*, *Col3a1*, *Fn1*, and *Acta2* and of *Tgfb1* was significantly suppressed in the UUO model following *Zdhhc18* KO (Figure 3M).

**Figure 4:**
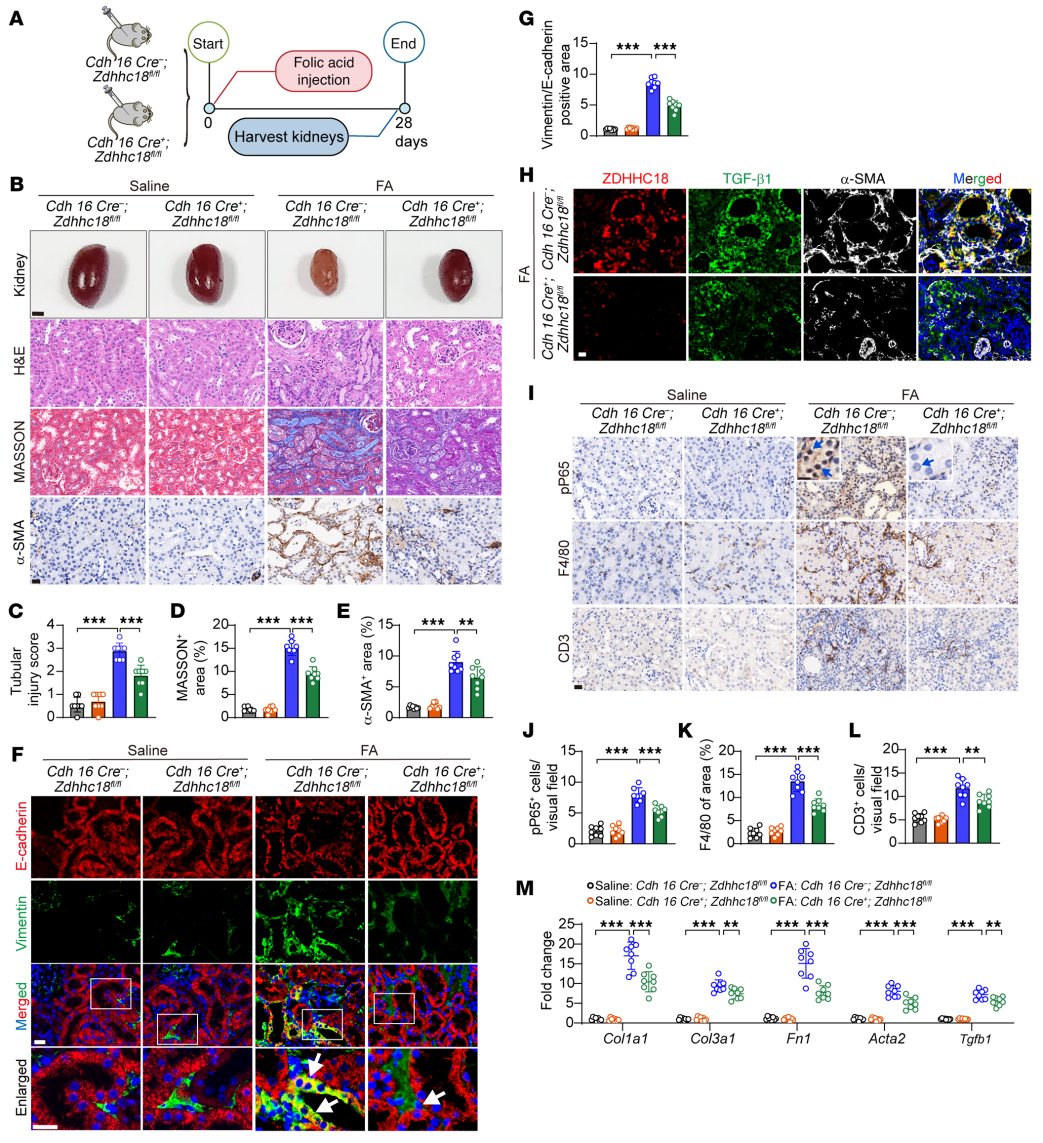
TEC-specific *Zdhhc18* deficiency inhibits renal fibrosis induced by FA in mice. (**A**) Experimental design. Harvesting kidneys of WT and *Zdhhc18*-CKO mice after saline or FA injection for 28 days. (**B**) Gross appearance of the kidneys (scale bar: 2 mm) as well as images of H&E, Masson, and α-SMA staining of WT and *Zdhhc18*-CKO mouse kidneys after FA. Scale bar: 20 μm. (**C**–**E**) Quantification of the tubular injury score (**C**), Masson staining of interstitial collagen (**D**), and α-SMA^+^ area (**E**) (*n* = 8). (**F**) Immunofluorescence images of staining. Square frame highlight digital enlargement of the tubule; white arrows indicate costaining of vimentin and E-cadherin. Scale bars: 20 μm. (**G**) Analysis of the percentage of vimentin- and E-cadherin–stained areas (*n* = 8). (**H**) Immunofluorescence images of ZDHHC18 (red), TGF-β1 (green), and α-SMA (white) expression in the kidneys of *Zdhhc18*-CKO mice after FA. Scale bar: 20 μm**.** (**I**) pP65, F4/80, and CD3 staining of WT and *Zdhhc18*-CKO mouse kidneys after FA. Blue arrow indicates pP65^+^ cells in the renal tubules. Scale bars: 20 μm (Enlarged: 10 μm). (**J**–**L**) Quantification of the proportion of pP65^+^ cells (**J**), F4/80^+^ area (**K**) and the proportion of CD3^+^ cells (**L**) (*n* = 8). (**M**) mRNA levels of fibrotic markers in kidneys of *Zdhhc18* CKO and WT mice (*n* = 8). Data are presented as the mean ± SD. **P < 0.01 and ***P < 0.001, by 2-way ANOVA with Tukey’s multiple-comparison test (**C**–**E**, **G**, and **J**–**M**).

We also used the FA model to investigate the role of *Zdhhc18* in kidney fibrosis (Figure 4A). The results of sCr and BUN measurements indicated that the severity of acute renal failure was not different between WT and *Zdhhc18*-KO mice on day 2 following FA administration (Supplemental Figure 6, A and B). On day 28 after FA administration, *Zdhhc18*-KO mice exhibited significantly improved renal function compared with WT mice, with lower sCr and BUN levels (Supplemental Figure 6, A–C). H&E, PAS, Masson, and Picrosirius red staining revealed that tubule-specific deletion of *Zdhhc18* ameliorated tubular atrophy and tubulointerstitial fibrosis in mice on day 28 after FA administration (Figure 4, B–D, and Supplemental Figure 6, D and E). Moreover, the number of interstitial α-SMA^+^ myofibroblasts was upregulated by FA treatment, but the upregulation of α-SMA was significantly reduced by *Zdhhc18* KO (Figure 4, B and E). Immunofluorescence analysis showed that *Zdhhc18* KO inhibited the FA-induced partial EMT process in renal TECs (Figure 4, F and G). Similar to the UUO model, *Zdhhc18* KO reduced TGF-β1 expression in renal tubules after FA and decreased the number of interstitial α-SMA^+^ myofibroblasts (Figure 4H). At day 28 after FA injection, *Zdhhc18*-CKO mice exhibited significantly reduced renal inflammation compared with WT mice, as evidenced by decreased mRNA levels of proinflammatory cytokines (Supplemental Figure 6F) and chemokines (Supplemental Figure 6G), along with attenuated nuclear phosphorylated NF-κB (pP65) in tubular cells, and reduced F4/80^+^ macrophages and CD3^+^ T cell infiltration (Figure 4, I–L). Ultimately, the expression of renal fibrosis markers in FA-induced *Zdhhc18*-KO mice was reduced (Figure 4M). In sum, data from the UUO and FA models demonstrate that TEC-specific KO *Zdhhc18* reduced renal fibrosis and inflammation in mouse CKD.

### Zdhhc18 overexpression exacerbates renal fibrosis.

**Figure 5:**
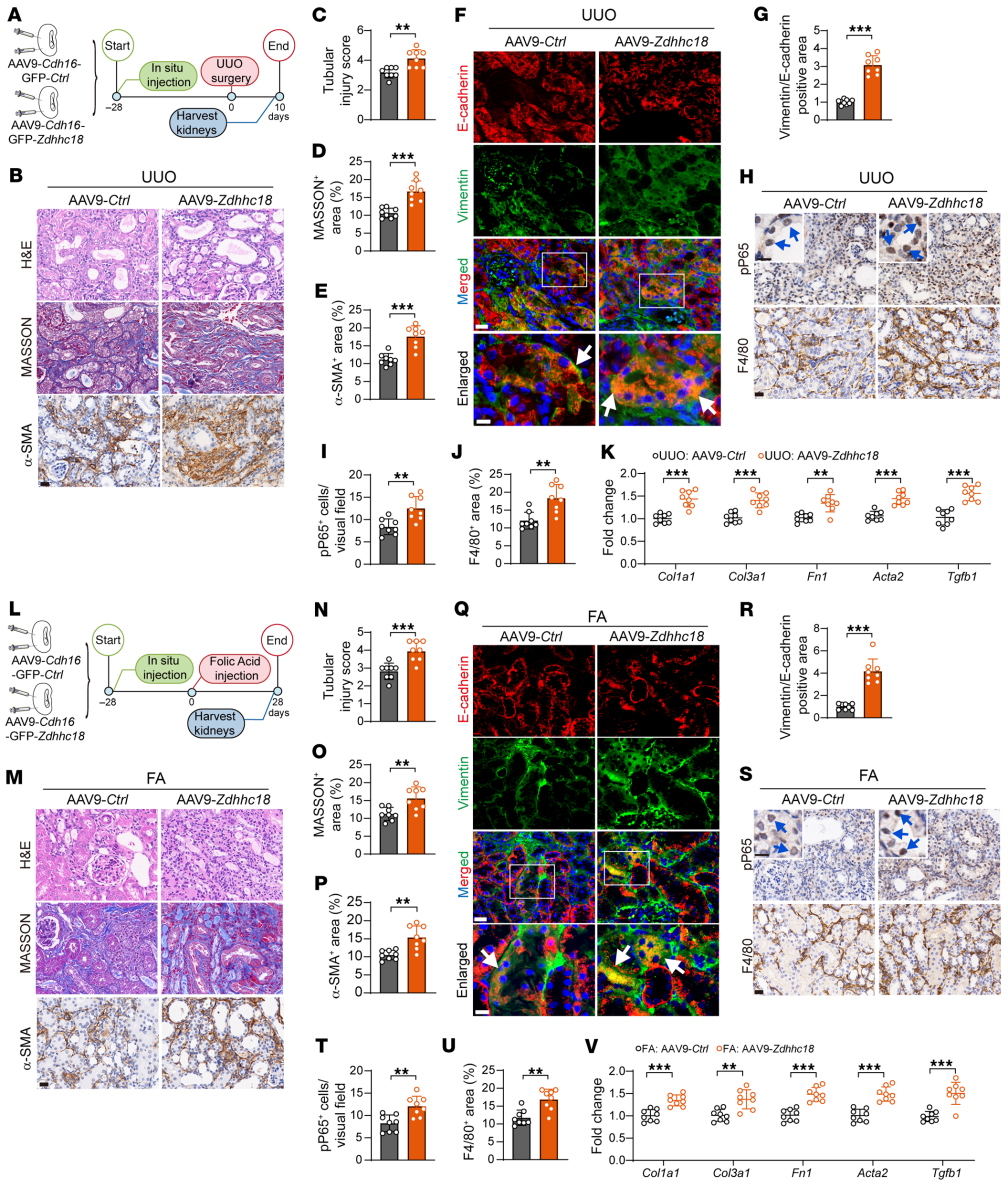
Overexpression of *Zdhhc18* exacerbates renal fibrosis induced by UUO and FA. (**A**) Experimental design. Mice were treated with multiple in situ injections into the kidney cortex. Four weeks later, they underwent UUO surgery, and the kidneys were harvested 10 days after surgery. (**B**) Images of H&E, Masson, and α-SMA staining of AAV9-*Ctrl* and AAV9-*Zdhhc18* kidneys. Scale bar: 10 μm. (**C**–**E**) Quantification of the tubular injury score (**C**) and Masson^+^ (**D**) and α-SMA^+^ (**E**) areas (*n* = 8). (**F**) Images of immunofluorescence staining. Square frames highlight digital enlargement of the tubule; white arrows point to vimentin and E-cadherin costaining. Scale bars: 20 μm. (**G**) vimentin and E-cadherin^+^ staining area ratio (*n* = 8). (**H**–**J**) Immunostaining of F4/80^+^ macrophages and pP65^+^ cells, with quantification (*n* = 8). The blue arrow indicates pP65^+^ cells in the renal tubules. (**K**) mRNA levels of fibrotic markers in UUO kidneys of AAV9-*Ctrl* and AAV9-*Zdhhc18* mice (*n* = 8). (**L**) Mice were treated with multiple in situ injections into the kidney cortex. Four weeks later, they underwent FA injection, and the kidneys were harvested 28 days later. (**M**–**P**) H&E, Masson, and α-SMA staining of AAV9-*Ctrl* and AAV9-*Zdhhc18* kidneys, with quantification (*n* = 8). (**Q**) Images of immunofluorescence staining. (**R**) Ratio of areas with vimentin^+^ and E-cadherin^+^ staining (*n* = 8). (**S**–**U**) Immunostaining of F4/80^+^ macrophages and pP65^+^ cells, with quantification (*n* = 8). (**V**) The mRNA levels of fibrotic markers in FA kidneys of AAV9-*Ctrl* and AAV9-*Zdhhc18* mice (*n* = 8). Scale bars: 20 μm (**B**, **F**, **H**, **M**, **Q**, and **S**) and 10 μm (enlarged insets in **H** and **S**). Data are presented as the mean ± SD. **P < 0.01 and ***P < 0.001, by 2-tailed Student’s *t* test (**C**–**E**, **G**, **I**–**K**, **N**–**P**, **R**, and **T**–**V**).

We used an adeno-associated virus (AAV) serotype 9 carrying the *Cdh16* (a kidney-specific cadherin exclusively expressed in TECs) promoter to overexpress GFP-tagged *Zdhhc18* in TECs. Four weeks after AAV injection, we found that GFP was expressed in the renal tubules but not in the glomeruli (Supplemental Figure 7A). IHC and qRT-PCR results showed that ZDHHC18 expression in the renal tubules was significantly increased (Supplemental Figure 7, B and C). Next, we analyzed AAV9-*Ctrl* and AAV9-*Zdhhc18* mice in the UUO kidney disease model (Figure 5A). *Zdhhc18* overexpression significantly exacerbated renal tubular injury and increased the number of interstitial α-SMA^+^ myofibroblasts and collagen deposition (Figure 5, B–E, and Supplemental Figure 7, D and E). Immunofluorescence analysis demonstrated that the increase in *Zdhhc18* overexpression accelerated the progression of UUO-induced partial EMT in TECs (Figure 5, F and G). Overexpression of *Zdhhc18* also increased the expression of proinflammatory cytokines (Supplemental Figure 7F) and chemokines (Supplemental Figure 7G), enhanced nuclear p-P65 expression in tubular cells, and increased infiltration of F4/80^+^ macrophages (Figure 5, H–J). qRT-PCR further confirmed that the expression of fibrosis markers was significantly upregulated in the UUO model following *Zdhhc18* overexpression (Figure 5K).

We also used the FA model to detect the function of *Zdhhc18* overexpression in renal fibrosis (Figure 5L). Indicators of kidney function, including sCr and BUN, were elevated in AAV9-*Zdhhc18* FA-treated mice compared with FA-injected AAV9-*Ctrl* mice (Supplemental Figure 7, H and I). *Zdhhc18* overexpression significantly exacerbated renal tubular injury and increased the number of interstitial α-SMA^+^ myofibroblasts and collagen deposition (Figure 5, M–P, and Supplemental Figure 7, J and K). Immunofluorescence analysis demonstrated that the increase in *Zdhhc18* expression accelerated the progression of FA-induced partial EMT in the kidneys (Figure 5, Q and R). Overexpression of *Zdhhc18* also increased the expression of proinflammatory cytokines (Supplemental Figure 7L) and chemokines (Supplemental Figure 7M), enhanced nuclear pP65 expression in tubular cells, and increased F4/80^+^ macrophage infiltration (Figure 5, S–U). qRT-PCR further confirmed that the expression of fibrosis markers was significantly upregulated in the FA model following *Zdhhc18* overexpression (Figure 5V). Collectively, these data demonstrate that *Zdhhc18* overexpression in renal TECs promotes renal fibrosis and inflammation in mouse CKD.

### ZDHHC18 controls HRAS palmitoylation and membrane localization.

**Figure 6:**
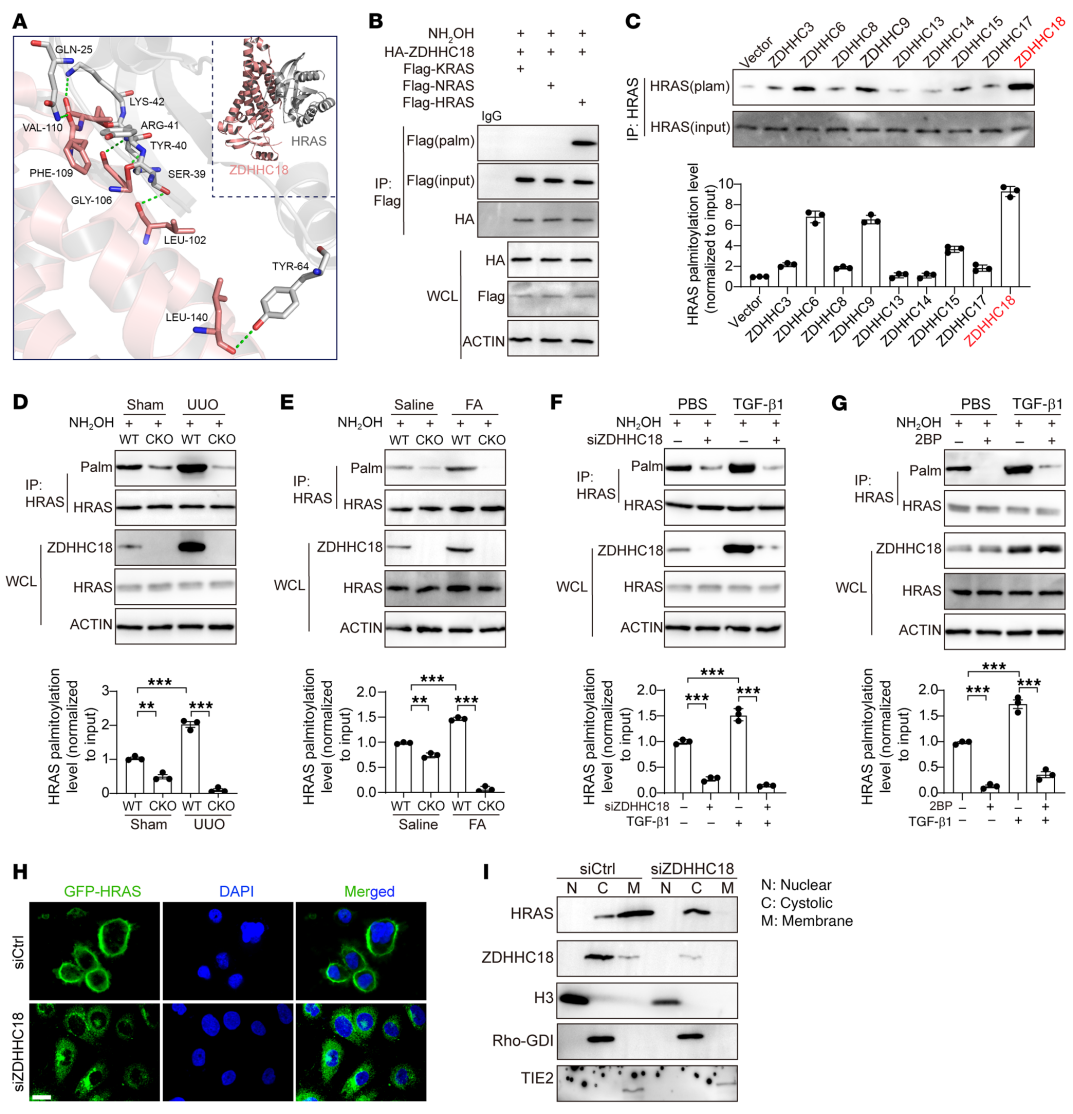
ZDHHC18-mediated HRAS palmitoylation regulates its plasma membrane localization. (**A**) Molecular docking simulations were performed on human ZDHHC18/HRAS to determine the strength of the interaction. (**B**) HK-2 cells were transfected with HA-ZDHHC18 and the Flag-RAS isoform for 48 hours. Cell lysates were collected for the ABE assay and immunoblot analysis. (**C**) HK-2 cells overexpressing ZDHHCs were subjected to an ABE assay and immunoblot analysis. (**D**) HRAS palmitoylation levels in the kidneys of WT and *Zdhhc18*-CKO mice were analyzed using ABE and immunoblot assays 10 days after UUO. (**E**) HRAS palmitoylation levels in the kidneys of WT and *Zdhhc18*-CKO mice were analyzed using ABE and immunoblot assays 28 days after FA. (**F**) HK-2 cells with *ZDHHC18* knockdown were treated with TGF-β1 for 48 hours. The palmitoylation status of HRAS was assessed using ABE and immunoblot assays. (**G**) HK-2 cells were treated with 2 μM 2BP for 48 hours and then stimulated with TGF-β1 for 48 hours. The palmitoylation status of HRAS was assessed using ABE and immunoblot assays. (**H**) Representative fluorescence images of GFP-HRAS staining in HK-2 cells transfected with si*ZDHHC18* or siCtrl. Scale bar: 50 μm. (**I**) Subcellular fractionation was performed on HK-2 cells transfected with siCtrl or si*ZDHHC18*, followed by immunoblot analysis using the indicated antibodies. For **C**–**G**, the results are representative of 3 independent biological experiments. HRAS palmitoylation levels were quantified using ImageJ software (**C**–**G**). WCL, whole-cell lysate; Palm, palmitoylation. Data are presented as the mean ± SD. **P < 0.01 and ***P < 0.001, by 2-way ANOVA with Tukey’s multiple-comparison test (**D**–**G**).

HRAS promotes partial EMT induced by TGF-β1, and KO of *Hras* reduces renal fibrosis caused by UUO (20–22). However, the specific molecular mechanisms by which HRAS exerts these effects are still unclear. We hypothesize that ZDHHC18 affects renal fibrosis through the palmitoylation of HRAS. To verify our hypothesis, molecular docking simulations were performed on human ZDHHC18/HRAS to determine the strength of the interaction. The simulation results showed that the binding energy between ZDHHC18 and HRAS was –17.6 kcal/mol (Figure 6A). Furthermore, to confirm the role of ZDHHC18 in RAS palmitoylation, we induced overexpression of ZDHHC18 and 3 isoforms of RAS (KRAS, NRAS, and HRAS) in HK-2 cells. We found that ZDHHC18 could bind to 3 types of RAS, but only HRAS underwent palmitoylation catalyzed by ZDHHC18 (Figure 6B). Prior research has indicated that RAS palmitoylation is catalyzed by ZDHHC6 and ZDHHC9 (24, 27). We induced overexpression of these 2 enzymes, as well as other ZDHHC enzymes that were upregulated in the UUO and FA models, in HK-2 cells. ZDHHC6, ZDHHC9, ZDHHC15, and ZDHHC18 promoted the palmitoylation of HRAS, and ZDHHC18 exhibited the highest upregulation (Figure 6C). ZDHHC6 and ZDHHC9 are downregulated during renal fibrosis (Figure 2A and Supplemental Figure 1B). These data indicate that the catalyst for HRAS palmitoylation during renal fibrosis was ZDHHC18. In mice, *Zdhhc18* KO did not affect the expression level of HRAS but did reduce the palmitoylation level of HRAS in the UUO- and FA-induced models (Figure 6, D and E). In HK-2 cells, an acyl-biotin exchange (ABE) assay showed that knocking down *ZDHHC18* or inhibiting ZDHHC18 activity through 2-BP reduced the palmitoylation of HRAS in TGF-β1–stimulated cells without affecting the expression level of HRAS (Figure 6, F and G). Silencing *ZDHHC18* with siRNA significantly reduced the membrane localization of HRAS in HK-2 cells (Figure 6H). We further assessed the subcellular localization of endogenous HRAS in siCtrl- and si*ZDHHC18-*treated HK-2 cells by using detergent-free subcellular fractionation assays. The markers for the different cellular fractions included RhoGDI for the cytosol, TIE2 for the membrane, and H3 for the nucleus. In si*Ctrl-*treated cells, HRAS was primarily located in the membrane fraction; however, in ZDHHC18-deficient cells, HRAS was located in the cytosolic fraction (Figure 6I). Collectively, these findings suggest that ZDHHC18 can associate with HRAS and facilitate its palmitoylation. Although palmitoylation did not affect the expression of HRAS, it did affect the membrane localization of HRAS.

### ZDHHC18 promotes partial EMT through the palmitoylation of HRAS.

**Figure 7:**
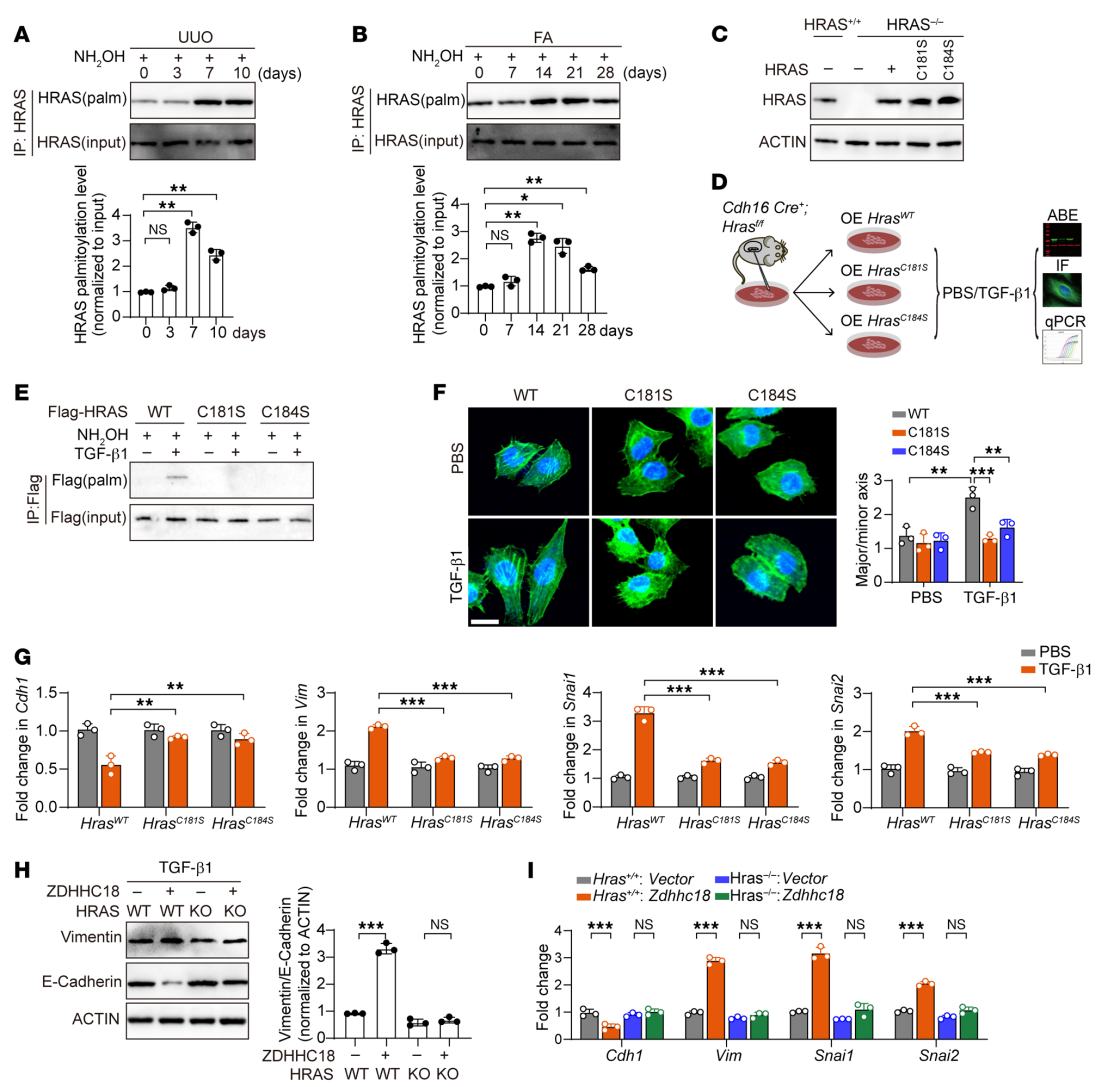
ZDHHC18 promotes partial EMT through the palmitoylation of HRAS. (**A** and **B**) HRAS palmitoylation levels in mouse kidneys after UUO (**A**) or FA (**B**) treatment were assessed by ABE assay with quantitative immunoblot analysis. (**C**) PTECs from WT and *Hras*-CKO mice were used to overexpress *Hras* (*Hras^WT^*) and its motif mutant C181S (*Hras^C181S^*) and C184S (*Hras^C184S^*). Immunoblot shows HRAS expression. (**D**) Experimental scheme. PTECs were isolated from the kidneys of *Cdh16 Cre^+^ Hras^fl/fl^* mice, which subsequently overexpressed *Hras^WT^*, *Hras^C181S^*, and *Hras^C184S^*. Then, the cells were stimulated with PBS or TGF-β1. (**E**) The palmitoylation status of HRAS was assessed using ABE and immunoblot assays. (**F**) Representative immunofluorescence confocal images of phalloidin-labeled cytoskeleton (green). Scale bar: 50 μm. (**G**) mRNA levels of partial EMT markers in PTECs were detected using qRT-PCR. (**H** and **I**) PTECs were isolated from the kidneys of WT and *Hras*-CKO mice and then transfected with an empty vector or a *Zdhhc18* overexpression construct. The PTECs were stimulated with TGF-β1. (**H**) Immunoblot of E-cadherin and vimentin expression in cells, with quantification of the blots. (**I**) mRNA levels of partial EMT markers in PTECs were detected using qRT-PCR. All data are representative of 3 independent biological experiments and are presented as the mean ± SD. *P < 0.05, **P < 0.01, and ***P < 0.001, by 1-way ANOVA with Tukey’s multiple-comparison test (**A** and **B**) and 2-way ANOVA with Tukey’s multiple-comparison test (**F**–**I**).

We further investigated the effect of HRAS palmitoylation on renal fibrosis. The results showed that in the renal fibrosis models induced by FA and UUO surgery, the palmitoylation levels of HRAS gradually increased (Figure 7, A and B). We generated mice with TEC-specific deletion of *Hras*. As expected, the kidneys of UUO *Hras*-CKO mice exhibited improved morphology with less damage than did the kidneys of the control mice subjected to UUO surgery (Supplemental Figure 8, A–D). *Hras* deficiency notably inhibited UUO-induced expression of the mesenchymal cell markers *Vim*, *Snai1*, and *Snai2* and upregulated *Cdh1* expression compared with expression levels in the control group (Supplemental Figure 8E). *Hras* KO also reduced the expression of fibrosis markers such as *Col1a1, Col3a1, Fn1*, and *Acta2* (Supplemental Figure 8F).

To further elucidate the mechanism, we isolated PTECs from *Hras*-KO mice and transfected them with WT HRAS (*Hras^WT^*), C181S-mutant HRAS (*Hras^C181S^*), and C184S-mutant HRAS (*Hras^C184S^*) (Figure 7, C and D). Computational analysis (https://swisspalm.org/) and existing articles (28, 29) suggested that cysteine residues at positions 181 and 184 were palmitoylation sites of HRAS. Interestingly, we found that TGF-β1 increased the palmitoylation of HRAS. Furthermore, the C181S and C184S mutations in HRAS almost completely abrogated HRAS palmitoylation (Figure 7E). Moreover, we observed that mutating the HRAS palmitoylation site in PTECs eliminated TGF-β1–induced morphological changes (Figure 7F). In addition to the TGF-β1–induced downregulation of epithelial cell marker expression, the upregulation of mesenchymal cell markers was inhibited by HRAS palmitoylation site mutations (Figure 7G). When *Hras* was knocked out, the downregulation of epithelial cell markers and the upregulation of mesenchymal cell markers triggered by ZDHHC18 overexpression was abolished in TECs (Figure 7, H and I). These data indicate that HRAS palmitoylation was required for TGF-β1–induced partial EMT and that ZDHHC18 promoted renal fibrosis in an HRAS palmitoylation–dependent manner.

### Palmitoylated HRAS-activated RREB1 recruits SMAD2/3 to the cis-regulatory regions of Snai1 and Has2.

**Figure 8:**
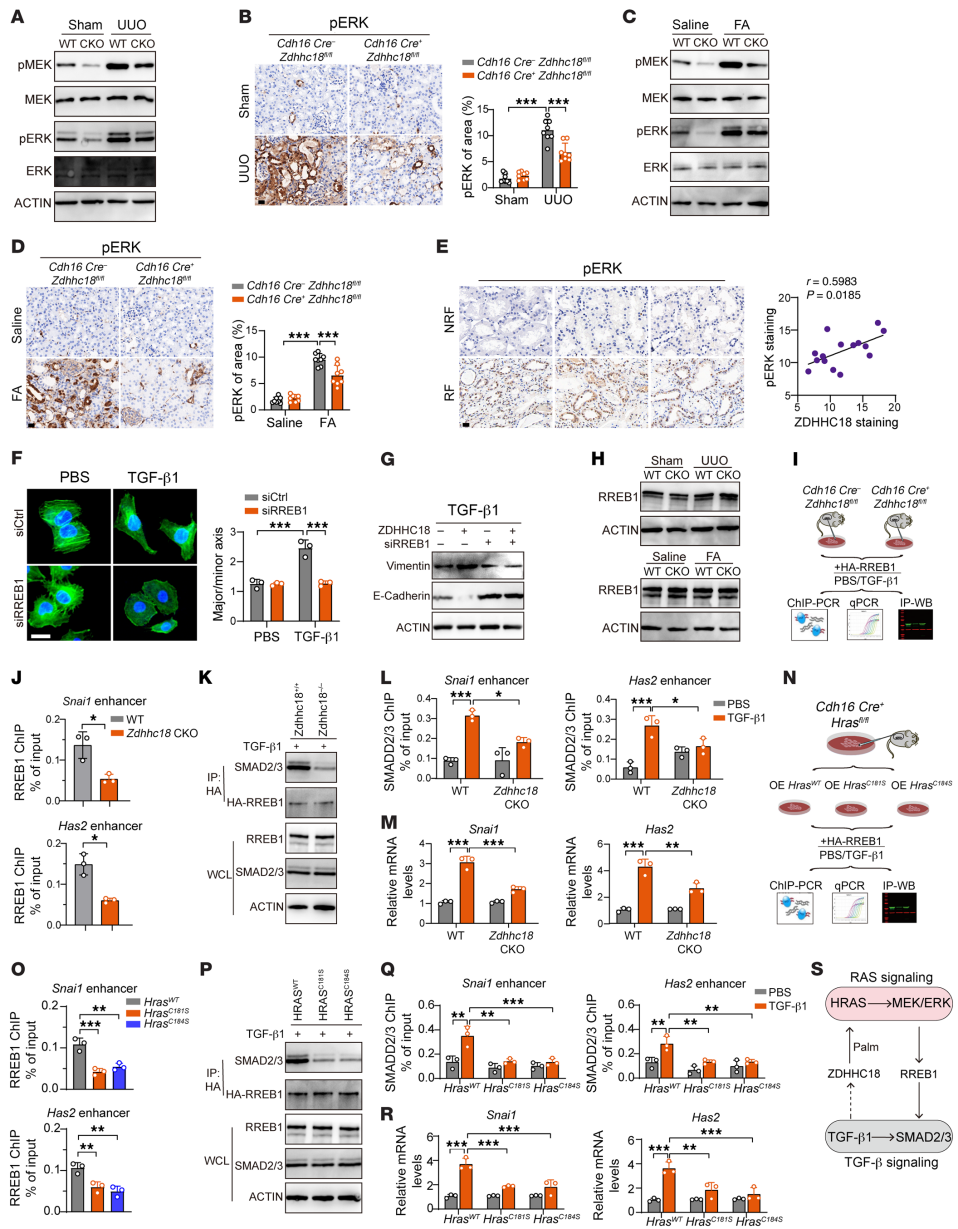
Palmitoylated HRAS–activated RREB1 recruits SMAD2/3 to the *cis*-regulatory regions of *Snai1* and *Has2*. (**A**) Immunoblot analysis of p-MEK, p-ERK, MEK, and ERK in WT and *Zdhhc18*-CKO mice after UUO. (**B**) p-ERK staining and quantification for UUO mice (*n* = 8). (**C**) Immunoblot analysis of p-MEK, p-ERK, MEK, and ERK after FA (*n* = 8). (**D**) p-ERK staining and quantification for FA mice (*n* = 8). (**E**) p-ERK staining for NRF and RF patients and Pearson’s correlation analysis between ZDHHC18 and p-ERK staining (*n* = 15). (**F** and **G**) PTECs were isolated from WT mice with *Rreb1* knockdown and treated with TGF-β1. (**F**) Confocal microscopy shows phalloidin (green) and DAPI (blue). Quantitative analysis of the major/minor axis of cells. (**G**) Immunoblotting for E-cadherin and vimentin expression in cells. (**H**) RREB1 immunoblot for WT and *Zdhhc18*-CKO mice after UUO and FA. (**I**–**M**) PTECs from WT and *Zdhhc18*-CKO mice were transfected with HA-RREB1 and treated with PBS/TGF-β1. (**J**) ChIP-PCR analysis of RREB1 binding to the enhancer regions of *Snai1* and *Has2*. (K) Cell lysates were collected for immunoprecipitation and immunoblot analysis. (**L**) ChIP-PCR analysis of SMAD2/3 binding to the enhancer regions of *Snai1* and *Has2*. (**M**) mRNA levels of *Snai1* and *Has2*. (**N**–**R**) PTECs were isolated from *Cdh16 Cre^+^* *Hras^fl/fl^* mice and overexpressed *Hras^WT^*, *Hras^C181S^*, and *Hras^C184S^*, followed by transfection with HA-RREB1 for 48 hours, with PBS or TGF-β1 stimulation. (**O**) ChIP-PCR analysis of RREB1 binding to the enhancer regions of *Snai1* and *Has2*. (**P**) Cell lysates were collected for immunoprecipitation and immunoblot analysis. (**Q**) ChIP-PCR analysis of SMAD2/3 binding to the enhancer regions of *Snai1* and *Has2*. (**R**) mRNA levels of *Snai1* and *Has2*. (**S**) Schematic of ZDHHC18-mediated RAS and TGF-β1 signaling. Scale bars: 20 μm (**B**, **D**, and **E**) and 50 μm (**F**). Data indicate the mean ± SD. *P < 0.05, **P < 0.01, and ***P < 0.001, by 1-way ANOVA with Tukey’s multiple-comparison test (**O**), by 2-way ANOVA with Tukey’s multiple-comparison test (**B**, **D**, **F**, **L**, **M**, **Q**, and **R**), and 2-tailed Student’s *t* test (**J**).

Next, we detected the activation of downstream signaling of HRAS. WB and IHC analyses revealed that the phosphorylated forms of MEK and ERK1/2 were increased in UUO and FA model mice and that p-MEK and p-ERK levels were significantly decreased in response to *Zdhhc18* depletion (Figure 8, A–D). Samples from patients with CKD also showed a positive correlation between ZDHHC18 staining levels and p-ERK staining intensity (Figure 8E). HRAS and SMAD2 were reported to promote EMT in tumor cells, but the mechanism of the interaction between these 2 proteins is not clear (30). Recent studies have shown that RAS/MEK/ERK signaling activates downstream RREB1 to promote TGF-β1 signaling (31). Here, we found that RREB1 knockdown in PTECs eliminated TGF-β1–induced morphological changes (Figure 8F) and abolished ZDHHC18 overexpression–mediated upregulation of partial EMT (Figure 8G). However, WB analysis showed that RREB1 expression was unchanged after knocking out *Zdhhc18* (Figure 8H). Research has demonstrated that ERK phosphorylation enhances RREB1 binding to the *cis*-regulatory regions of *Snai1* and *Has2* genes. These *cis*-regulatory elements are located in enhancer regions, where RREB1 binding promotes the expression of *Snai1* and *Has2* (31). *Snai1* and *Has2* are considered to promote EMT (32, 33). Next, we induced overexpression of HA-labeled RREB1 in PTECs (Figure 8, I and N). The ChIP-PCR results indicated that knocking out *Zdhhc18* or mutating the palmitoylation site of *Hras* weakened the binding of HA-RREB1 to the enhancer regions of *Snai1* and *Has2* (Figure 8, J and O). Coimmunoprecipitation revealed that the binding of SMAD2/3 to HA-RREB1 was reduced by knocking out *Zdhhc18* or mutating the palmitoylation site of *Hras* (Figure 8, K and P). Knocking out *Zdhhc18* or mutating the palmitoylation site of *Hras* also weakened the binding of SMAD2/3 to the enhancer regions of *Snai1* and *Has2* (Figure 8, L and Q). The induction of these genes by TGF-β1 was attenuated by knocking out *Zdhhc18* or mutating the *Hras* palmitoylation site (Figure 8, M and R). Collectively, our findings demonstrate that ZDHHC18-mediated palmitoylation of HRAS activated downstream RREB1, which was associated with TGF-β1–induced SMAD2/3 binding and the promotion of SMAD2/3 activity. TGF-β1 also promoted the expression of ZDHHC18. This highlights ZDHHC18’s key role in linking TGF-β and RAS signaling pathways (Figure 8S).

## Discussion

ZDHHC18 is known to function as a ZDHHC-palmitoyl transferase. However, its specific substrates have not been fully characterized. ZDHHC18 has been reported to catalyze the palmitoylation of MDH2 and promote the development of ovarian cancer (34). ZDHHC18 has also been recognized as a negative regulator of cGAS activity, thereby mitigating the innate immune response (35). However, its role in renal fibrosis has not been explored. Our study demonstrated a marked increase in ZDHHC18 levels in fibrotic kidneys from both humans and mice. We identified ZDHHC18 as a promoter of renal fibrosis by using CKO mice. Mechanistically, ZDHHC18 catalyzed HRAS palmitoylation, thereby upregulating MEK/ERK signaling. This activated RREB1, a RAS-responsive transcription factor, which enhanced SMAD2/3 binding to *Snai1* and *Has2* *cis*-regulatory regions, facilitating TGF-β1–induced partial EMT.

Through sci-RNA-Seq data and immunofluorescence analysis, we found that the upregulation of ZDHHC18 in the kidney was mainly concentrated in PT-FR. Subsequently, we found that knocking out *Zdhhc18* in renal TECs markedly inhibited UUO- and FA-induced renal fibrosis. Endothelial cells play a crucial role in renal fibrosis (36, 37). sci-RNA-Seq results showed that the expression of *Zdhhc18* in endothelial cells was also slightly upregulated during UUO. Further investigation of the specific contribution of endothelial ZDHHC18 to renal fibrosis progression would provide valuable insights into its cell-type–specific functions and therapeutic potential.

We found consistent cytokine expression responses in the kidney during UUO- and FA-induced renal fibrosis. In vitro experiments showed that *Zdhhc18* KO inhibited certain cytokine expressions in PTECs, whereas in vivo studies demonstrated reduced macrophage and T cell infiltration. During fibrosis, these cytokines are released by TECs undergoing partial EMT, fibroblasts, and infiltrating immune cells. Our data suggest that *Zdhhc18* KO reduced renal inflammation by inhibiting partial EMT in TECs. EMTs are driven by *Snail*, *Zeb*, and *Twist* transcription factors (32, 38). Two previous studies showed that partial EMT of epithelial cells induced by *Snail1* and *Twist1* promotes inflammatory responses during renal fibrosis (26, 39). However, questions remain about which cytokines are secreted by each cell subtype after partial EMT and their proportional contributions. These questions warrant more precise answers through single-cell sequencing of TEC-specific KO mice with classic EMT-driven genes (such as *Snail1* and *Twist1*).

While previous studies identified ZDHHC9 as being responsible for RAS palmitoylation (28, 40, 41), recent research has indicated that other ZDHHCs, such as ZDHHC6, can also mediate RAS palmitoylation (27). Our data indicate that HRAS palmitoylation increased during renal fibrosis. We found that ZDHHC6, ZDHHC9, and ZDHHC18 could catalyze HRAS palmitoylation but that the expression of ZDHHC6 and ZDHHC9 was downregulated during renal fibrosis. Consequently, we propose that ZDHHC18 plays a critical role in HRAS palmitoylation during renal fibrosis. This hypothesis is supported by the results shown in Figures 6 and 7, in which ZDHHC18 KO resulted in a reduction in HRAS palmitoylation.

RAS palmitoylation has significant physiological importance (42, 43). Prior studies have demonstrated that a mutation in the NRAS palmitoyl site can rescue the myeloid-transformed phenotype that is upregulated by the NRAS^G12D^-activating mutation (44). In our study, we found an increase in HRAS palmitoylation in mice with UUO- or FA-induced renal fibrosis. These findings were further substantiated by our investigations using PTECs from *Hras*-KO mice that overexpressed *Hras* harboring mutations at palmitoylation sites 181 and 184. These mutations were shown to alleviate the partial EMT phenotype. To elucidate the specific contributions of HRAS palmitoylation to renal fibrosis, future studies could use mice with targeted genomic mutations at HRAS C181S and HRAS C184S and model fibrosis in these animals.

The ZDHHC family of palmitoyltransferases includes 23 members, each capable of modifying various substrates involved in identical physiological processes. Even a single substrate can be targeted by multiple palmitoyltransferases, leading to divergent outcomes. For instance, NLRP3 can undergo palmitoylation by both ZDHHC5 and ZDHHC12; ZDHHC5 enhances NLRP3 activation (45), whereas ZDHHC12 promotes NLRP3 degradation and inhibits its function (46). Therefore, it is impossible to accurately determine the role of palmitoylation modification of all proteins in a certain process such as renal fibrosis. At the same time, the use of inhibitors such as 2-BP cannot accurately inhibit the palmitoylation of a certain protein without affecting other important proteins that are palmitoylated during this process. Furthermore, given that palmitoylation is reversible (47), the identification of acyl protein thioesterases (APTs) and their specific roles (including those that may act on HRAS) remains a critical area for research. One study reported that *Apt1* is upregulated during renal fibrosis and that knocking out *Apt1* inhibits renal fibrosis (15). However, we analyzed the RNA-Seq data and did not observe any significant changes in the expression of APT family members during renal fibrosis (Figure 2A).

Many articles have reported that RAS promotes TGF-β/SMAD signaling (30, 48–50), but the mechanism by which RAS promotes TGF-β1–induced EMT was not discovered until recently. Continuously activated KRAS signaling in pancreatic cancer cells activates RREB1, which promote the binding of SMAD2/3 to the *cis*-regulatory regions of target genes (31). In our study, we demonstrated that HRAS activated RREB1, enhancing SMAD2/3 association with *Snai1* and *Has2* *cis*-regulatory regions during renal fibrosis. Notably, we discovered that ZDHHC18-mediated HRAS palmitoylation was requisite for RREB1 activation. Concurrently, research investigating the effect of TGF-β signaling on RAS signaling pathways is scarce. In this study, we found that TGF-β1 could promote the expression of ZDHHC18, thereby promoting the palmitoylation-mediated activation of HRAS. These results allow us to understand how RAS and TGF-β signaling activates each of these factors during renal fibrosis to promote partial EMT and exacerbate fibrosis. We also showed that ZDHHC18 is a key molecule involved in the communication between the 2 signaling pathways, suggesting that ZDHHC18 is a therapeutic target for the treatment of renal fibrosis.

Overall, our study indicates that ZDHHC18 contributed to partial EMT in TECs by catalyzing HRAS palmitoylation, which enhanced the association of RREB1 with *Snai1* and *Has2* *cis*-regulatory regions. This RREB1 interaction amplified SMAD2/3 activity.

## Methods

Additional details on methods can be found in the Supplemental Methods.

### Sex as a biological variable.

Human kidney biopsy samples were obtained from both men and women. Our CKD mouse model exclusively examined male mice to reduce female sexual cycle–related variation. It is unknown whether the findings in male mice are relevant to female mice.

### Human renal biopsy samples.

Renal biopsy specimens were collected from the Department of Nephrology at Guangzhou Women and Children’s Medical Center, Guangzhou Medical University. Control samples (*n* = 8) were obtained from patients who underwent diagnostic biopsies for hematuria but showed no pathological alterations. The renal fibrosis group (*n* = 15) was identified based on Masson staining. All specimens underwent histological analysis with quantitative scoring. Detailed patient information and scoring criteria are available in Supplemental Table 1 and Supplemental Methods.

### Mice.

CRISPR/Cas9 technique was used to generate *Zdhhc18^fl/+^* and *Hras^fl/+^* mice on a C57BL6/J background by Suzhou Cyagen Co. *Cdh16*-*Cre*–transgenic mice were obtained from Cyagen (C001022; C57BL/6J background). Floxed *Zdhhc18* mice Cyagen (S-CKO-11349; C57BL/6J background) or floxed *Hras* mice (Cyagen no. S-CKO-02970; C57BL/6J background) were hybridized with transgenic mice expressing *Cre*-recombinase under the *Cdh16* promoter to specifically knock out *Zdhhc18* or *Hras* in renal TECs (Tub- *Zdhhc18^–/–^*, genotype: *Cre*^+/–-^, *Zdhhc18^fl/fl^*, or Tub- *Hras^–/–^*, genotype: *Cre^+/–^*, *Hras^fl/fl^*). Control littermate mice were sex-matched *Zdhhc18^fl/fl^* *Cre^–/–^* or *Hras^fl/fl^* *Cre^–/–^* mice from the same litters.

### Statistics.

Statistical analyses were conducted using GraphPad Prism 9 (GraphPad Software). Data are presented as the mean ± SD. For comparisons between 2 groups, a 2-tailed Student’s *t* test was applied. For multiple group comparisons, 1-way ANOVA followed by Tukey’s test was used, whereas 2-way ANOVA with Tukey’s test was used for analyses involving multiple variables. Pearson’s correlation analysis was performed to determine correlation coefficient *r* and *P* values, and linear regression was used to assess relationships between variables. A *P* value of less than 0.05 was considered statistically significant. All statistical details are provided in the main and Supplemental Figure legends.

### Study approval.

All clinical sample collection procedures were approved by ethics committee of Guangzhou Women and Children’s Medical Center. All participants were duly informed, and written consent was obtained from the patients. Animal experiments were approved by the IACUC of Guangdong Huawei Testing Co., Ltd.

### Data availability.

The authors declare that all data supporting the findings of this study are available in the main text or the supplemental material, including the Supporting Data Values file. The publicly available mouse renal transcriptomics data and single-cell combinatorial indexing RNA-Seq data used in this study are available in the Gene Expression Omnibus (GEO) database (GSE125015, GSE65267, and GSE190887).

## Author contributions

QW and YJ conceived the project and supervised the study. QW, YJ, and XG contributed to the conception and design of the study and helped revise the manuscript. DL and GA designed and performed the major experiments, analyzed data, and interpreted the results. YL, GA, SJ, RY, SZ, and GL provided technical support. QW wrote the manuscript, and all the other authors discussed and formulated the manuscript. WF and DL contributed to the collection and analysis of clinical specimens. The order of the first authors’ names was determined on the basis of their contributions to the work.

## Supplementary Material

Supplemental data

Unedited blot and gel images

Supporting data values

## Version 1 02/06/2025

In-Press Preview

## Version 2 03/17/2025

Electronic publication
