# Supplementary material for: ZDHHC18 promotes renal fibrosis development by regulating HRAS palmitoylation
Source: J Clin Invest. 2025 Feb 4;135(6):e180242. doi: 10.1172/JCI180242 (PMC11910235; doi:10.1172/JCI180242)
Supplement: Supplemental data [file jci-135-180242-s014.pdf]

Supplemental Information

ZDHHC18 promotes renal fibrosis development by regulating HRAS palmitoylation

Di Lu[1†], Gulibositan Aji[2†], Guanyu Li[1], Yue Li [1], Wenlin Fang[3], Shuai Zhang[4], Ruiqi Yu[5], Sheng Jiang[2], Xia Gao[1*], Yuhang Jiang[6*] and Qi Wang[7*]

Inventory for Supplemental Information

I. Supplemental methods

II. References

III. Supplemental Figure

IV. Supplemental Table

Supplemental methods

UUO model

Eight-week-old male mice were anesthetized with pentobarbital sodium (30 mg/kg) by an intraperitoneal injection. The back was opened, and the left ureter was ligated with 4-0 silk. The left ureter is mobilized, ligated with silk sutures at both proximal and distal points, and transected between the ligations. The back tissue was closed, and the skin was sutured. After surgery, the animals were kept on a warm pad to maintain a constant body temperature. The sham-operated group was not subjected to ligation.

FA model

Eight-week-old male mice were intraperitoneally injected with 250 mg/kg folic acid (#HY-16637, MedChemExpress) dissolved in 0.3 M sodium bicarbonate. Equal volumes of 0.3 M sodium bicarbonate were injected into mice in the vehicle control group. The mice were euthanized after 4 weeks. Kidneys were collected for further analysis.

AAV delivery

An adeno-associated virus (AAV) 9 system was used to deliver Zdhhc18 to C57BL/6J mice. The mouse Zdhhc18 was cloned downstream of the kidney-specific *Cdh16* promoter (1341 bp, nucleotides 2430-770, GenBank accession no. AF118228) in AAV9 vector (1, 2). GFP was linked for tracking expression. Briefly, C57BL/6J mice (males, aged 4 weeks) were anesthetized with pentobarbital sodium (30 mg/kg) by intraperitoneal injection, and were injected

with 2 × 10^{12} vector genome (vg)/ml AAV9-*Cdh16*-GFP-*Zdhhc18* and 2 × 10^{12} vg/ml AAV9-*Cdh16*-GFP control into 5 different sites (10 μl at each site) of the renal cortex with a glass micropipette. Experiments were performed 4 weeks post AAV injection.

Histopathology

Kidney tissues were fixed in 4% paraformaldehyde for at least 24 hours, embedded in paraffin and sectioned (3μm). Sections were deparaffinized and rehydrated through graded alcohols according to standard procedures. For H&E staining, sections were stained with hematoxylin for 5 minutes, differentiated in 1% acid alcohol, and blued in lithium carbonate. Sections were then counterstained with eosin for 1 minute. For Periodic Acid-Schiff (PAS) staining, sections were oxidized with periodic acid solution for 10 minutes, rinsed with distilled water, then stained with Schiff reagent (#BBL-0213, Biossci) for 15 minutes and washed under running tap water for 10 minutes. For MASSON staining, sections were immersed in Bouin's solution (#BP005, Biossci) overnight, stained with iron hematoxylin (#BBL-0200, Biossci) for 10 minutes, differentiated in 1% hydrochloric acid alcohol, and blued in lithium carbonate. Sections were then stained with Ponceau S (10 minutes), treated with phosphomolybdic acid (5 minutes), and counterstained with aniline blue (5 minutes). For Sirius red staining, a modified Sirius red staining kit (#G1078, Servicebio) was used according to the manufacturer's instructions.

Immunohistochemistry and immunofluorescence staining

Kidney sections were deparaffinized, rehydrated, and subjected to heat-induced antigen retrieval. After blocking with 2.5% goat serum, sections were incubated with primary antibodies (ZDHHC18, Vimentin, α-SMA, pP65, F4/80, CD3, pERK; dilutions listed in Supplemental Table 3) at 4°C overnight. Following PBS washes, sections were incubated with secondary antibodies and developed using DAB chromogen. Sections were counterstained with hematoxylin, dehydrated through an ethanol gradient, cleared in xylene, and mounted using neutral resin. Images were captured using a Servicebio Case Viewer scanner.

For kidney tissue immunofluorescence staining, sections were permeabilized with 0.1% Triton X-100 and blocked with 5% goat serum in PBS for 20 minutes at room temperature. Sections were then incubated with primary antibodies overnight at 4°C, followed by fluorophore-conjugated secondary antibodies. Nuclei were counterstained with DAPI. For cells, formaldehyde was used for fixation for 15 min, and permeabilized with 0.2% Triton X-100 for 15 minutes. Then, the slides were immunostained with primary antibodies. Fluorescently labeled secondary antibodies were used. The samples were stained with DAPI (#C0065, Solarbio) and observed. Samples were analyzed, and pictures were taken using Leica SP8 confocal scanning microscope. Image J is used for immunofluorescence quantitative analysis. Detailed information about primary antibodies, including manufacturers, catalog numbers, and working dilutions, is provided in Supplemental Table 3.

Tubular injury score

For human renal biopsy samples tubular injury was assessed on H&E-stained according to the following scoring system (3): tubular epithelial cell flattening (1 point), brush border loss (1 point), cell membrane bleb formation (1-2 points), interstitial edema (1 point), cytoplasmic vacuolization (1 point), cell necrosis (1-2 points), and tubular lumen obstruction (1-2 points). For mouse tubular injury was assessed on H&E-stained according to the following scoring system (4): 0 (no injury), 1 (1-20% affected area), 2 (21-50% affected area), 3 (51-75% affected area), and 4 (>75% affected area). All samples evaluated by two independent pathologists who examined renal cortical tubules from 5 random, non-overlapping fields per kidney sample under high-power microscopy (400×). All assessments were performed in a blinded manner.

Pearson correlation analysis

The relative expression levels of ZDHHCs and fibrosis-related genes were determined by quantitative real-time PCR. For each sample, the relative expression value was calculated using the formula 2^ (Ct GAPDH - Ct target), where Ct target represents the average cycle threshold of either ZDHHCs or fibrosis-related genes, and Ct GAPDH represents the average cycle threshold of GAPDH as the internal reference gene. The correlation between ZDHHCs and fibrosis-related genes was analyzed by plotting their relative expression values on scatter plots using GraphPad Prism software (version 8.0). Pearson's correlation coefficient (*r*) was calculated to assess the strength and direction of

the relationship between gene pairs, with $p < 0.05$ considered statistically significant.

Bioinformatic analysis

Pre-processed sci-RNA-seq data and metadata were downloaded from GEO (GSE190887). Quality control excluded cells with: n_feature_counts >6000 or <200, >20% mitochondrial counts, and >15% ribosomal gene counts. Highly variable genes were identified using VST method. After log-transformation and scaling, PCA was performed for dimension reduction. Data integration used scvi-tools (v0.20.3) with sample_id, n_Feature_RNA, and n_Count_RNA as covariates. The 20-dimensional latent representation was visualized using UMAP. Clusters were identified using Leiden clustering (resolution=1.0). Cell-type markers were determined using Wilcoxon Rank-Sum test with multiple testing correction. Clusters were annotated based on marker genes and compared with original annotations. Zdhhc18 expression was visualized using sc.pl.dotplot.

The bulk RNA-seq data of UUO and FA models were downloaded from GEO Datasets (GSE125015, GSE65267). The online OmicShare heatmap tool (http://www.omicshare.com/tools) was used to draw the expression heat map of ZDHHCs based on the gene expression value. ZDHHCs whose expression is zero are not included in the heat map.

HK-2 in vitro studies

Human proximal tubular epithelial cells (HK-2) were obtained from Procell

(Wuhan, China) and cultured in DMEM-F12 medium (#A4192002, Gibco™) supplemented with 10% FBS (cat: A5670701, Gibco™) and 1% penicillin-streptomycin (#15140148, Gibco™) at 37°C with 5% CO_2. Culture medium was replaced every 48 hours.

For *ZDHHC18* knockdown, HK-2 cells were seeded in 6-well plates and grown to 80-90% confluence. Cells were transfected with 20 pM si*ZDHHC18* or non-targeting control siRNA using Lipofectamine 3000 (#L3000001, Invitrogen) following manufacturer's instructions. The siRNA sequences are listed in Supplementary Table 2. Knockdown efficiency was validated after 48 hours by RT-qPCR and Western blot analysis of ZDHHC18 expression. For overexpression studies, HK-2 cells at 80-90% confluence were transfected with pcDNA3.1-ZDHHC18, pcDNA3.1-HRAS, pcDNA3.1-NRAS, or pcDNA3.1-KRAS expression plasmids (Tsingke Biotech, Guangzhou, China) using Lipofectamine 3000. Overexpression was confirmed after 48 hours by RT-qPCR and Western blot analysis. Following 48 hours of transfection (either siRNA or plasmid), cells were treated with 20 ng/ml TGF-β1 (#100-21, PeproTech) for an additional 48 hours before analysis.

Primary tubular epithelial cell isolation and in vitro experiments

Primary tubular epithelial cells were isolated as described previously with several modifications (5, 6). The mouse was anesthetized and the abdominal cavity was opened to expose the thoracic aorta. After perfusion of 10 ml of magnetic beads (#14011, Invitrogen) at a concentration of $4x10^6$ beads/ml PBS

through the thoracic aorta, the kidneys were removed and the renal capsule. The renal cortex was isolated and minced into 1-3 mm^3 fragments. Tissue fragments were then digested in 1 mg/ml type V collagenase (#C8170, Solarbio) at 37°C for 15 min. The digested tissue was filtered and placed on a magnetic separation rack to collect glomeruli. The resulting suspension was passed through a 100 μm cell strainer to remove undigested tissue, followed by a 40 μm cell strainer to eliminate single cells. The tubules retained on the 40 μm cell strainer were collected for further analysis. After washing 3 times with PBS, tubules were plated onto culture dishes pre-coated with type I collagen (5 $\mu g/cm^2$). Cells were maintained in renal epithelial growth medium consisting of DMEM/F12 supplemented with 10% fetal bovine serum, 1% penicillin-streptomycin, and 10 ng/mL epidermal growth factor. Cultures were incubated at 37°C in a humidified atmosphere of 5% CO_2. The initial medium change was performed at 72 hours post-plating, followed by medium renewal every 48 hours thereafter.

To generate *Hras* mutant PTECs, the plvx-*Hras*^{C181S} or plvx-*Hras*^{C184S} construct (Tsingke Biotech, Guangzhou, China) was incorporated into lentiviruses and used to infect passage 3 PTECs isolated from *Hras* CKO mice. For overexpression studies, PTECs at 80-90% confluence was transfected with pcDNA3.1-*Rreb1* expression plasmids (Tsingke Biotech, Guangzhou, China) using Lipofectamine 3000. Overexpression was confirmed after 48 hours by RT-qPCR and Western blot analysis. Following 48 hours of transfection, cells

were treated with 20 ng/ml TGF-β1 (#100-21, Peprotech) for an additional 48 hours before analysis.

Immunoprecipitation

Whole-cell lysates were harvested after being transfected or stimulated and were incubated overnight with anti-Flag beads (#FFP19, Beyotime), anti-HA beads or HRAS (#Sc-35, Santa Cruz) antibodies plus protein A/G beads (#HYK0242, MCE). The beads were washed three times with wash buffer (#P2175S, Beyotime), and the immunoprecipitates were resuspended in loading buffer and boiled at 100 °C for 10 min.

Immunoblotting

We used RIPA buffer (#P0013C, Beyotime) to lyse tissues or cell pellets. The samples were incubated on ice for at least ten minutes to ensure proper cell lysis. A Lowry protein assay was used to calculate the protein concentration. The proteins were subsequently separated by SDS–PAGE. The proteins were transferred to a PVDF membrane (#FFP19, Beyotime). The designated proteins were examined by immunoblotting with specific antibodies in 5% bovine serum albumin. The antibodies used are listed in Supplemental Table 3.

Acyl-biotin exchange palmitoylation assay

The cells were lysed with lysis buffer (1% NP-40, 150 mM NaCl, 50 mM Tris-HCl (pH 7.5), 10% glycerol, and protease inhibitors) supplemented with 50 mM N-ethylmaleimide for 1 h at 4 °C, after which the endogenous HRAS or Flag-tagged HRAS were purified with specific antibodies or beads. The purified

HRAS protein was treated with freshly prepared HAM-containing buffer (50 mmol/L Tris-HCl (pH 7.2), 1% NP-40, 10% glycerol, 150 mmol/L NaCl, and 1 mol/L HAM) and incubated at room temperature for 1 hour. Finally, the beads were gently washed with lysis buffer (pH 6.2) and incubated with lysis buffer (pH 6.2) plus 2 μM biotin-BMCC (#21900, Thermo Fisher Scientific) at 4 °C for 1 hour. The samples were ultimately washed three times and subjected to immunoblot analysis.

Cell fractionation assay

First, 5×10^7 HK-2 cells were resuspended in 2 mL of hypotonic homogenization buffer (10 mM KCl, 1.5 mM MgCl2, and 10 mM HEPES at pH 7.4) and kept on ice for 10 minutes. The cells were homogenized approximately 20 times using a Dounce homogenizer with a tightly fitting pestle, and lysis was confirmed by microscopy. The nuclei were collected by centrifugation at 500g for 10 minutes at 4 °C, sonicated in 3 mL of SDS loading buffer, and subsequently boiled for 10 minutes at 95 °C. The postnuclear supernatant was transferred to a polycarbonate tube and subjected to ultracentrifugation at 350,000 ×g for 1 hour at 4 °C. The supernatant was collected as the cytosolic fraction, and the pellet was lysed in 2 mL of 1% NP-40 lysis buffer for 30 minutes at 4 °C to isolate the membrane fraction. SDS loading buffer (1 mL) was added to the cytosolic and membrane fractions, which were subsequently boiled for 10 minutes at 95 °C. All the fractions were then subjected to WB analysis.

RNA extraction and real-time RT-PCR

A TaKaRa MiniBEST Universal RNA Extraction Kit (#9767, TaKaRa) was used to extract total RNA. For tissue samples, A total of 20 mg of kidney tissue was homogenized in 600μl of Buffer RL with tissue lyzer (Wonbio-P) for 1min at 4°C. For cultured cells, cells were directly lysed in the culture dish by adding 600μl of Buffer RL per well of 6-well plate after removing the culture medium and washing with PBS. After lysis, samples were incubated at room temperature for 2 min and mixed with an equal volume of 70% ethanol. The mixture was transferred to an RNA spin column and centrifuged at 12,000 rpm for 1 min. The column was washed with Buffer RWA and Buffer RWB. Finally, the RNA was eluted with RNase-free water. RNA was pretreated with DNase before proceeding to cDNA conversion. A total of 1.5μg RNA was converted into cDNA using the PrimeScript™ RT Master Mix (#RR036A, TaKaRa). Conduct real-time quantitative PCR performed with the TB Green® Premix Ex Taq™ (#RR420A TaKaRa) using the CFX PCR System (Bio-Rad, CA). Analysis to measure relative gene expression by normalizing the CT value of the target gene to that of the reference gene (*Gapdh was used*). Data are calculated and expressed as fold changes using the $2^{-\Delta\Delta CT}$ method. Primer sequences are listed in Supplemental Table 4.

ChIP–PCR analysis

A total of 1×10^6 cells were fixed with formaldehyde for 10 min and sonicated with UCD-300 (Bioruptor). Antibody binding was reversed by cross-linking using

an EZ-ChIP immunoprecipitation kit (#17-371, Millipore). The antibodies used for ChIP were rabbit monoclonal anti-SMAD2/3 (#8685, CST) and rabbit monoclonal anti-HA-Tag (#3724, CST). The primer sequences (5′-3′) used for PCR amplification are listed in Supplemental Table 5.

References

1. Shao X, Johnson JE, Richardson JA, Hiesberger T, and Igarashi P. A minimal Ksp-cadherin promoter linked to a green fluorescent protein reporter gene exhibits tissue-specific expression in the developing kidney and genitourinary tract. *J Am Soc Nephrol.* 2002;13(7):1824-36.
2. Asico LD, Cuevas S, Ma X, Jose PA, Armando I, and Konkalmatt PR. Nephron segment-specific gene expression using AAV vectors. *Biochem Biophys Res Commun.* 2018;497(1):19-24.
3. Tian SF, Jiang ZZ, Liu YM, Niu X, Hu B, Guo SC, et al. Human urine-derived stem cells contribute to the repair of ischemic acute kidney injury in rats. *Mol Med Rep.* 2017;16(4):5541-8.
4. Nlandu-Khodo S, Osaki Y, Scarfe L, Yang H, Phillips-Mignemi M, Tonello J, et al. Tubular beta-catenin and FoxO3 interactions protect in chronic kidney disease. *JCI Insight.* 2020;5(10).
5. Zhang D, Xing Y, Li W, Yang F, Lang Y, Yang J, et al. Renal tubules transcriptome reveals metabolic maladaption during the progression of ischemia-induced acute kidney injury. *Biochem Biophys Res Commun.* 2018;505(2):432-8.

6. Cao X, Wang J, Zhang T, Liu Z, Liu L, Chen Y, et al. Chromatin accessibility dynamics dictate renal tubular epithelial cell response to injury. *Nat Commun.* 2022;13(1):7322.

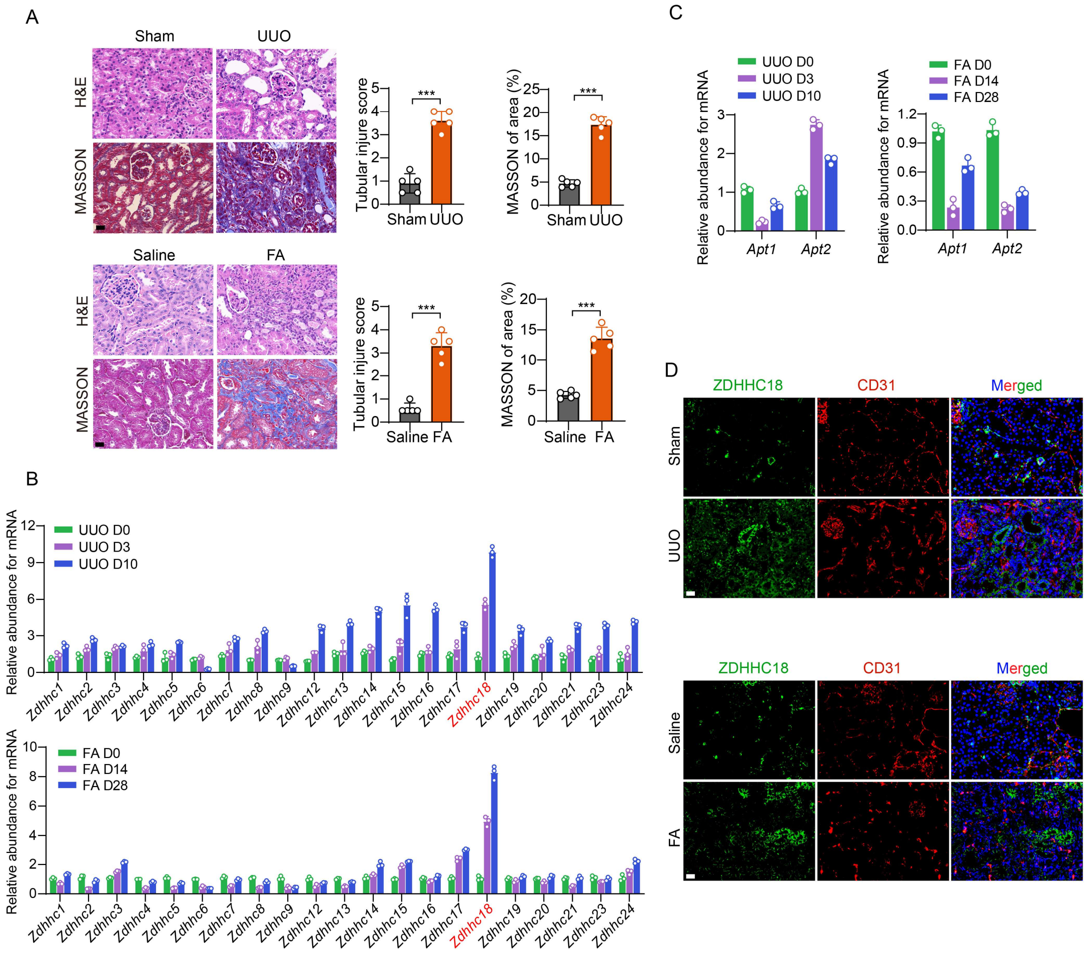

Supplemental Figure 1. The expression of *Zdhhc18* was increased in the kidneys of mice with fibrosis. (A) H&E and MASSON staining of kidneys after 10 days of UUO and 28 days of FA. Scale bar: 20 μm. Data are presented as the mean±SD.**P* < 0.05, ***P* < 0.01, ****P* < 0.001, by two-tailed Student's t test. (**B**) The mRNA levels of *ZDHHC* family members in mouse kidney after UUO (0, 3, and 10 days) and FA (0, 14, and 28 days) (n=3). (**C**) The mRNA levels of *Apt1* and *Apt2* in the kidneys of mice at different days after UUO and FA (n=3). (**D**) Confocal microscopy staining of ZDHHC18 (green), CD31 (red), and DAPI (blue) in UUO and FA. Scale bar: 20 μm.

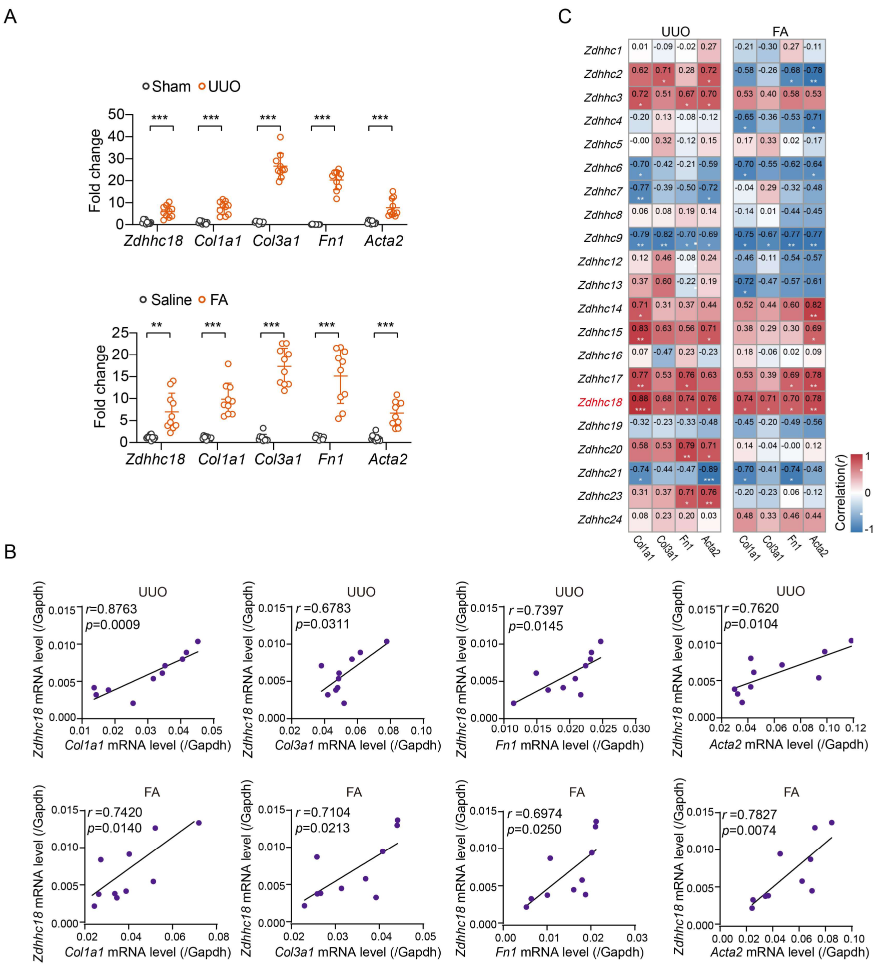

Supplemental Figure 2. Positive correlation between *Zdhhc18* mRNA expression and fibrotic markers in kidneys of fibrotic mice. (**A**) *Zdhhc18*, *Col1a1*, *Col3a1*, *Fn1* and *Acta2* mRNA levels in the kidneys of mice after 10 days of UUO and 28 days of FA (n=10). Data are presented as the mean±SD.**P* < 0.05, ***P* < 0.01, ****P* < 0.001, by two-tailed Student's t test. (**B**) Pearson's correlation between *Zdhhc18* and fibrosis marker mRNA expression after UUO and FA (n=10). (**C**) Pearson's correlation analysis of mRNA expression between ZDHHC family members and fibrotic markers following UUO and FA treatment (n=10). The correlation matrix shows coefficients (r) ranging from -1 to 1, where red and blue hues indicate positive and negative correlations respectively, with color intensity proportional to correlation strength. Statistical significance: *p < 0.05, **p < 0.01, ***p < 0.001.

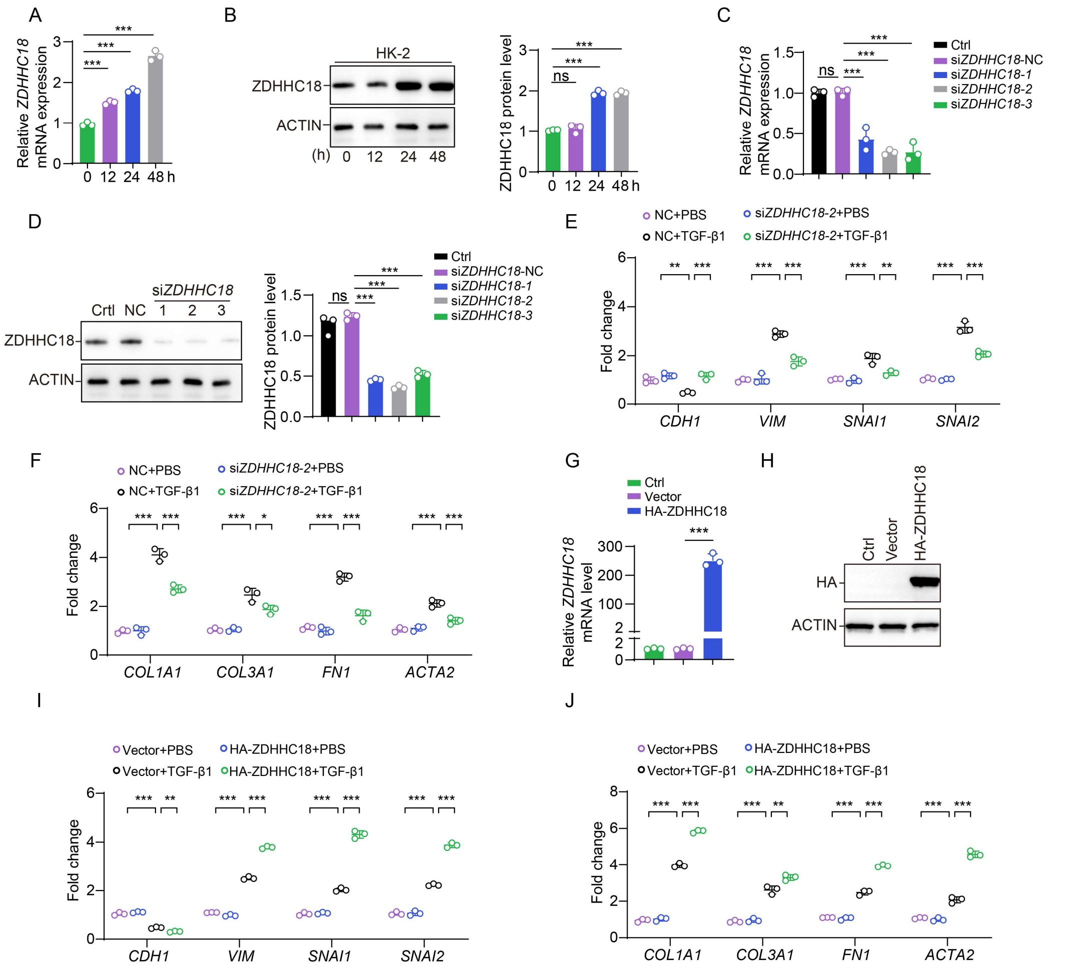

Supplemental Figure 3. *ZDHHC18* was responsible for the TGF-β1-induced alterations in HK-2 cells. (**A**) The levels of *ZDHHC18* mRNA were measured after stimulation with TGF-β1 for different times. (**B**) The protein levels of ZDHHC18 were analyzed by Western blotting (WB) after stimulation with TGF-β1 for different times. Quantification of ZDHHC18 protein by ImageJ. (**C**) *ZDHHC18* mRNA levels after knockdown in HK-2 cells. Ctrl, blank control. si*ZDHHC18*-NC, negative control. si*ZDHHC18-1*, si*ZDHHC18-2* and si*ZDHHC18-3* were the three different siRNA sequences of *ZDHHC18*. (**D**) ZDHHC18 protein levels detected by WB after knockdown in HK-2 cells. Quantification of ZDHHC18 WB by ImageJ. (**E** and **F**) HK-2 cells with or without *ZDHHC18* knockdown were stimulated with TGF-β1. The mRNA levels of partial EMT markers (**E**) and fibrotic markers (**F**) were analyzed using qRT-PCR. (**G**) *ZDHHC18* mRNA levels in HK-2 cells overexpressing *ZDHHC18*. Vector, transfection with an empty vector. HA-ZDHHC18, transfected with the full-length ZDHHC18 plasmid. (**H**) HA-ZDHHC18 protein levels detected by WB after overexpressing in HK-2 cells. (**I** and **J**) HK-2 cells with or without *ZDHHC18* overexpressing were stimulated with TGF-β1. The mRNA levels of partial EMT markers (**I**) and fibrotic markers (**J**) were analyzed using qRT-PCR. Data are presented as the mean±SD.*$P < 0.05$, **$P < 0.01$, ***$P < 0.001$, by one-way ANOVA with Tukey's multiple-comparison test (**A-D**, **G** and **H**) and by two-way ANOVA with Tukey's multiple-comparison test (**E**, **F**, **I** and **J**). ns, not significant. The data was a representative of multiple experiments.

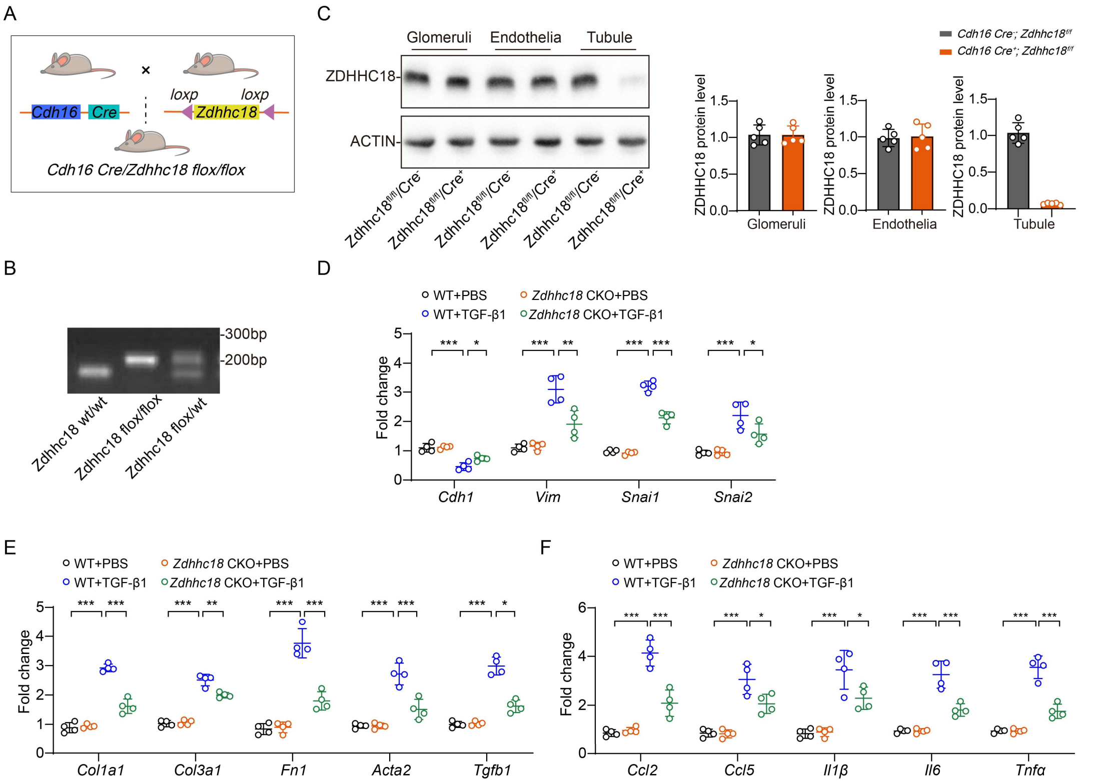

Supplemental Figure 4. Generation of tubule-specific *Zdhhc18* knockout (*Cdh16 Cre*$^{+}$; *Zdhhc18*$^{f/f}$) mice. (**A**) Schematic of the strategy used to generate *Zdhhc18* conditional knockout (CKO) mice. (**B**) The mice were genotyped by PCR analysis of genomic DNA as indicated. (**C**) Isolation of glomeruli, endothelia and tubules from *Zdhhc18* CKO mice and WB analysis to determine the protein expression levels of ZDHHC18, with quantification of bands compared to the ACTIN control. (**D-F**) The primary tubular epithelial cells (PTECs) of WT and *Zdhhc18* CKO were stimulated with TGF-β1. qRT-PCR detection of partial EMT markers (**D**) and fibrotic markers (**E**) in PTECs (n=4). (**F**) The mRNA levels of chemokines (*Ccl2*, *Ccl5*), inflammatory cytokines (*Il1β*, *Il6*, *Tnfα*) in PTECs (n=4). Data are presented as the mean±SD.*$P < 0.05$, **$P < 0.01$, ***$P < 0.001$, by two-way ANOVA with Tukey's multiple-comparison test (**D-F**).

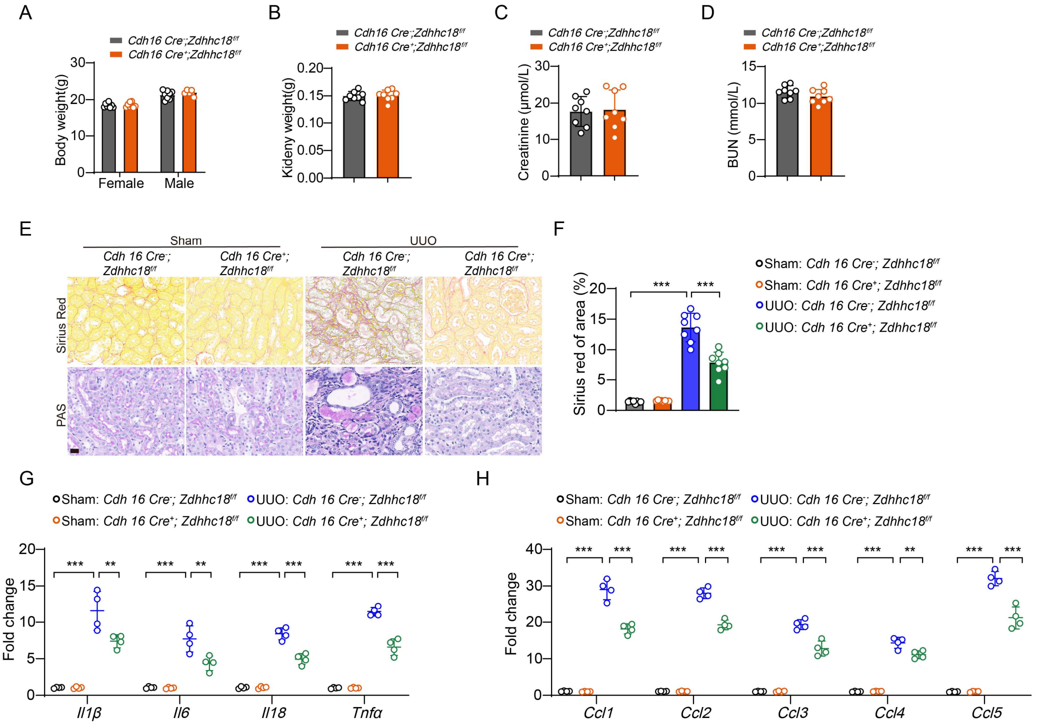

Supplemental Figure 5. Specific deficiency of *Zdhhc18* in TECs attenuates renal fibrosis induced by UUO in mice. (**A-D**) Graphs showing the body weight (**A**), kidney weight (**B**), blood creatinine (**C**), and BUN (**D**) in the knockout mice and control littermates 2 months after birth (n=8). (**E**)The kidneys of WT and *Zdhhc18* CKO mice were subjected to Sirius Red staining and PAS staining on the 10 days after UUO. Scale bar: 20 μm. (**F**) Quantification of Sirius Red-positive area (n=8). (**G** and **H**) The mRNA levels of inflammatory cytokines (*Il1β*, *Il6*, *Il18*, *Tnfα*) (**G**) and chemokines (*Ccl1*, *Ccl2*, *Ccl3, Ccl4*, *Ccl5*) (**H**) in the kidneys of WT and *Zdhhc18* CKO mice after Sham and UUO (n=4). Data are presented as the mean±SD.*$P < 0.05$, **$P < 0.01$, ***$P < 0.001$, by two-way ANOVA with Tukey's multiple-comparison test.

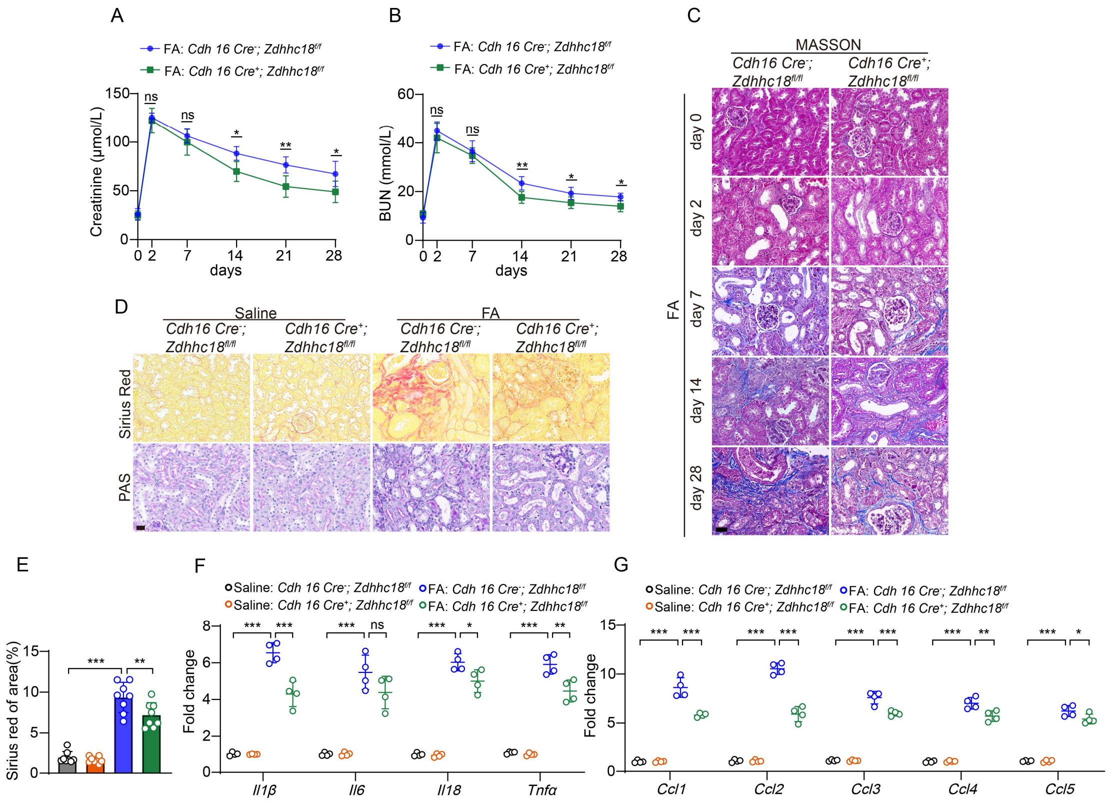

Supplemental Figure 6. Specific deficiency of *Zdhhc18* in TECs attenuates of renal fibrosis induced by FA in mice. (**A** and **B**) Dynamic changes of *Zdhhc18* CKO during kidney injury and repair in FA model. Changes of creatinine (**A**) and BUN (**B**) in groups as indicated (n=5). (**C**) Representative images of mice kidney samples from groups as indicated stained with MASSON. Scale bar: 20 μm. (**D**)The kidneys of WT and *Zdhhc18* CKO mice were subjected to Sirius Red and PAS staining 28 days after FA. (**E**) Quantification of Sirius Red-positive area (n=8). Scale bar: 20 μm. (**F** and **G**) The mRNA levels of inflammatory cytokines (*Il1β*, *Il6*, *Il18*, *Tnfα*) (**F**) and chemokines (*Ccl1*, *Ccl2*, *Ccl3, Ccl4*, *Ccl5*) (**G**) in the kidneys of WT and *Zdhhc18* CKO mice after Saline and FA (n=4). Data are presented as the mean±SD.**P* < 0.05, ***P* < 0.01, ****P* < 0.001, by two-tailed Student's t test (**A** and **B**) and by 2-way ANOVA with Tukey's multiple-comparison test (**E-G**).

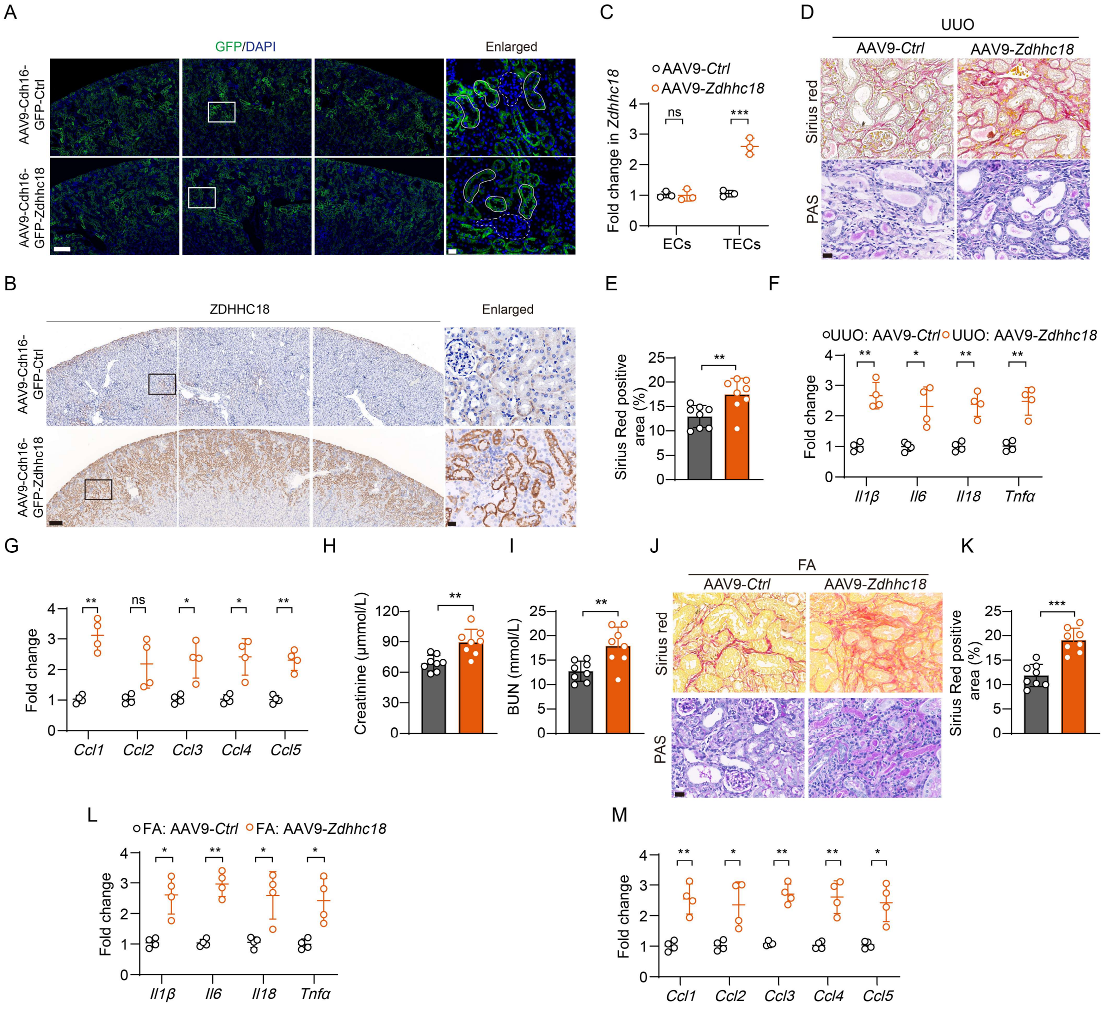

Supplemental Figure 7. Overexpression of *Zdhhc18* exacerbates renal fibrosis induced by UUO and FA. (**A**) Representative images of GFP fluorescence in the renal cortex of mice injected with AAV9-*Cdh16*-GFP-*Ctrl* and AAV9-*Cdh16*-GFP-*Zdhhc18* (Solid line: renal tubule, dotted line: glomerulus). Scale bar: 200 μm (Enlarged: 20 μm). (**B**) Immunohistochemistry of ZDHHC18 in renal cortex. Scale bar: 200 μm (Enlarged: 20 μm). (**C**) *Zdhhc18* mRNA levels were detected in isolated endothelial and tubular epithelial cells from AAV9-*Ctrl* and AAV9-*Zdhhc18* groups. (**D** and **E**) Histological staining of Sirius Red and PAS in kidney sections and quantitative analysis of AAV9-*Ctrl* and AAV9-*Zdhhc18* mice 10 days after UUO surgery (n = 8). Scale bar: 20 μm. (**F** and **G**) The mRNA levels of inflammatory cytokines (**F**) and chemokines (**G**) in the kidneys of AAV9-*Zdhhc18* and AAV9-*Ctrl* mice after UUO (n = 4). (**H** and **I**) Analysis of serum creatinine (**H**) and BUN (**I**) in AAV9-*Ctrl* and AAV9-*Zdhhc18* mice at FA 28 days. (**J** and **K**) Histological staining of Sirius Red and PAS in kidney sections and quantitative analysis of AAV9-*Ctrl* and AAV9-*Zdhhc18* mice 28 days after FA (n = 8). Scale bar: 20 μm. (**L** and **M**) The mRNA levels of inflammatory cytokines (**L**) and chemokines (**M**) in the kidneys of AAV9-*Zdhhc18* and AAV9-*Ctrl* mice after FA (n = 4). Data are presented as the mean±SD.**P* < 0.05, ***P* < 0.01, ****P* < 0.001, by two-tailed Student's t test.

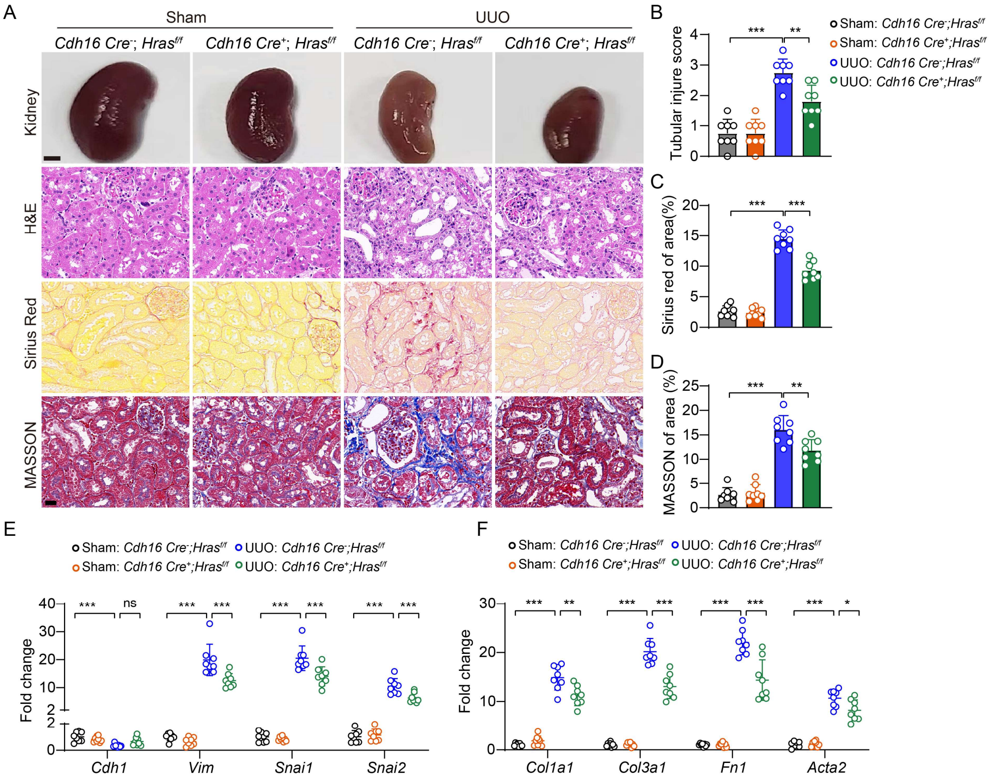

Supplemental Figure 8. Specific knockout *Hras* in TECs alleviated UUO-induced renal fibrosis in mice. (**A**)The gross appearance of kidneys (Scale bar: 2 mm) and H&E staining, Sirius Red staining, and MASSON staining of WT and *Hras* CKO mice 10 days after UUO surgery. Scale bar: 20 μm. (**B-D**) Quantification of the tubular injury score (**B**), Sirius Red-positive area (**C**), and fibrosis-positive area (**D**) (n=8). (**E** and **F**) The mRNA levels of partial EMT markers(**E**) and fibrotic markers (**F**) in Sham and UUO kidneys of *Hras* CKO and WT mice(n=8). Gene expression levels were normalized to *Gapdh*. Data are presented as the mean±SD.**P* < 0.05, ***P* < 0.01, ****P* < 0.001, by 2-way ANOVA with Tukey's multiple-comparison test.

Supplemental Table 1. Clinical information of the subjects

Normal group(n=8)	
Age(years)	11.8±1.7
Gender(male,n,%)	3(37.5%)
SCr(μmol/l)	68.6±25.5
BUN (mmol/l)	5.3±2.5
eGFR(ml/min/1.73m^2)	110.6±42.1
Subjects with renal fibrosis(n=15)	
Age(years)	12.4±3.1
Gender(male,n,%)	7(41.1%)
SCr (μmol/l)	109.7±42.3
BUN (mmol/l)	13.3±4.1
eGFR(ml/min/1.73m^2)	43.1±20.4
Pathological diagnosis	N (%)
Focal segmental glomerulosclerosis	3(20%)
Lupus nephritis	5(33.33%)
IgA nephropathy	2(13.33%)
Renal tubulointerstitial lesions	1(6.66%)
Henoch-Schonlein Purpura Nephritis (HSPN)	4(26.68%)

Number	Group	Pathological diagnosis	Gender	Age	SCr	BUN	eGFR
1	NRF	-	Male	11	63.35	5.56	179.34
2	NRF	-	Male	10	58.88	3.38	158.55
3	NRF	-	Male	13	65.55	4.34	123.37
4	NRF	-	Female	12	60.08	4.05	93.32
5	NRF	-	Female	11	130.33	10.43	53.35
6	NRF	-	Female	10	55.87	3.32	87.97
7	NRF	-	Female	15	64.76	3.89	76.76
8	NRF	-	Female	12	49.67	7.83	112.35
9	RF	Focal segmental glomerulosclerosis	Female	7	120.24	20.08	34.54
10	RF	Lupus nephritis	Female	12	153.68	21.22	24.53
11	RF	Focal segmental glomerulosclerosis	Female	8	201.2	14.54	37.54
12	RF	Lupus nephritis	Female	15	115.52	12.45	28.09
13	RF	Lupus nephritis	Female	15	87.78	10.98	66.87
14	RF	Lupus nephritis	Female	16	179.76	15.23	20.54
15	RF	HSPN	Female	12	58.55	8.76	39.76
16	RF	HSPN	Female	16	60.55	6.78	71.43
17	RF	Focal segmental glomerulosclerosis	Male	9	153.68	18.84	17.65
18	RF	Lupus nephritis	Male	14	77.65	8.78	84.65
19	RF	HSPN	Male	15	80.77	10.11	69.09
20	RF	IgA nephropathy	Male	10	90.03	11.15	50.09
21	RF	IgA nephropathy	Male	9	70.98	15.49	19.9
22	RF	HSPN	Male	16	99.19	12.34	36.54
23	RF	Renal tubulointerstitial lesions	Male	12	95.66	12.22	44.65

Supplemental Table 2. List of siRNA

Gene name	Sequences
si*ZDHHC18*-1	Sense: AAUACUUGAAACGGGUUUATT
	Antisense: UAAACCCGUUUCAAGUAUUTT
si*ZDHHC18*-2	Sense: UGUAUCAGACAAAGGUAAATT
	Antisense: UUUACCUUUGUCUGAUACATT
si*ZDHHC18*-3	Sense: UUAUUCUCUCCCUCUCAUUTT
	Antisense: AAUGAGAGGGAGAGAAUAATT

Gene name	Sequences
si*Rreb1*-1	Sense: ACACACACUGGUAAGAAGGCCCUCA
	Antisense: UGAGGGCCUUCUUACCAGUGUGUGU
si*Rreb1*-2	Sense: ACCAGGCGGUGAGUCUUGAAAGGAA
	Antisense: UUCCUUUCAAGACUCACCGCCUGGU
si*Rreb1*-3	Sense: GGUGAGUCUUGAAAGGAAAGAGUAA
	Antisense: UUACUCUUUCCUUUCAAGACUCACC

Supplemental Table 3. List of Primary antibodies

Primary antibodies	Source	Provider	Catalog	Application
ZDHHC18	Rabbit	Novus	NBP1-85057	IF (1:200), IHC (1:500)
α-SMA-Cy3	Mouse	Merck	C6198	IF 1:200
TGFβ1-488	Rabbit	Abcam	AB313729	IF 1:50
Vimentin	Rabbit	Abways	CY5134	IF (1:100), IHC (1:100), WB (1:1000)
E-cadherin	Mouse	CST	14472	IF (1:200), WB (1:1000)
VCAM1-647	Rabbit	Bioss	0396R	IF (1:200)
CD31-488	Rabbit	CST	66477	IF (1:200)
phosphor-P65	Rabbit	CST	3037	IHC (1:50)
F4/80	Rabbit	Abcam	AB111101	IHC (1:100)
CD3	Rabbit	Abcam	AB135372	IHC (1:100)
α-SMA	Mouse	Abcam	AB7817	IHC (1:500)
pERK	Rabbit	CST	4370	IHC (1:200) WB (1:1000)
ZDHHC18	Rabbit	Abcam	AB154790	WB (1:5000), IHC (1:500)
ACTIN	Rabbit	Abclonal	AC026	WB (1:10000)
HRAS	Rat	Santa Cruz	SC-35	WB (1:500)
Rho-GDI	Mouse	Santa Cruz	SC-365190	WB (1:500)
Flag	Rabbit	CST	2368	WB (1:1000)
HA	Rabbit	CST	3724	WB (1:1000), ChIP(1:50)
H3	Rabbit	CST	9715	WB
TIE2	Mouse	CST	4224	WB (1:500)
MEK	Rabbit	CST	9122	WB (1:1000)
pMEK	Rabbit	CST	9154	WB (1:1000)
ERK	Rabbit	CST	9102	WB (1:1000)
SMAD2/3	Rabbit	CST	8685	WB (1:1000), ChIP(1:100)
RREB1	Rabbit	GenwayBiotech	5B0668	WB (1:1000)

Supplemental Table 4. List of primers used for qRT-PCR

Gene names	Primer	Sequence
Human *ZDHHC18*	Forward	5’-GCAACTTCCTCTCCACTCTG-3’
Human *ZDHHC18*	Reversed	5’-GTACGTGTGAAACCCTGAGAG-3’
Human *COL1A1*	Forward	5’-CCCCTGGAAAGAATGGAGATG-3’
Human *COL1A1*	Reversed	5’-TCCAAACCACTGAAACCTCTG-3’
Human *COL3A1*	Forward	5’-AAGTCAAGGAGAAAGTGGTCG-3
Human *COL3A1*	Reversed	5’-CTCGTTCTCCATTCTTACCAGG-3
Human *SNAIL1*	Forward	5’-GGAAGCCTAACTACAGCGAG-3’
Human *SNAIL1*	Reversed	5’-CAGAGTCCCAGATGAGCATTG-3’
Human *SNAIL2*	Forward	5’-AGCATTTCAACGCCTCCA-3’
Human *SNAIL2*	Reversed	5’-GGATCTCTGGTTGTGGTATGAC-3’
Human *FN1*	Forward	5’-ACTGTACATGCTTCGGTCAG-3’
Human *FN1*	Reversed	5’-AGTCTCTGAATCCTGGCATTG-3’
Human *ACTA2*	Forward	5’-AATGCAGAAGGAGATCACGG-3’
Human *ACTA2*	Reversed	5’-TCCTGTTTGCTGATCCACATC-3’
Human *VIM*	Forward	5’-CGTGAATACCAAGACCTGCTC-3’
Human *VIM*	Reversed	5’-GGAAAAGTTTGGAAGAGGCAG-3’
Human *CDH1*	Forward	5’-CCCAATACATCTCCCTTCACAG-3’
Human *CDH1*	Reversed	5’-CCACCTCTAAGGCCATCTTTG-3’
Human *GAPDH*	Forward	5’-ACATCGCTCAGACACCATG-3’
Human *GAPDH*	Reversed	5’-TGTAGTTGAGGTCAATGAAGGG-3’
Mouse *Zdhhc18*	Forward	5’-CAACTTCCTCTCCGCTCTG-3’
Mouse *Zdhhc18*	Reversed	5’-TCTTCGTTAGTTGTCAGGTTGG-3’
Mouse *Col1a1*	Forward	5’-CATAAAGGGTCATCGTGGCT-3’
Mouse *Col1a1*	Reversed	5’-TTGAGTCCGTCTTTGCCAG-3’
Mouse *Col3a1*	Forward	5’-GAAGTCTCTGAAGCTGATGGG-3’
Mouse *Col3a1*	Reversed	5’-TTGCCTTGCGTGTTTGATATTC-3’
Mouse *Fn1*	Forward	5’-CTTTGGCAGTGGTCATTTCAG-3’
Mouse *Fn1*	Reversed	5’-ATTCTCCCTTTCCATTCCCG-3’
Mouse *Snai1*	Forward	5’-ACATCCGAAGCCACACG-3’
Mouse *Snai1*	Reversed	5’-GTCAGCAAAAGCACGGTTG-3’
Mouse *Snai2*	Forward	5’-ACACATTAGAACTCACACTGGG-3’
Mouse *Snai2*	Reversed	5’-TGGAGAAGGTTTTGGAGCAG-3’
Mouse *Vim*	Forward	5’-TTTCTCTGCCTCTGCCAAC-3’
Mouse *Vim*	Reversed	5’-TCTCATTGATCACCTGTCCATC-3’
Mouse *Acta2*	Forward	5’-GTGAAGAGGAAGACAGCACAG-3’
Mouse *Acta2*	Reversed	5’-GCCCATTCCAACCATTACTCC-3’
Mouse *Tgfb1*	Forward	5’-CCTGAGTGGCTGTCTTTTGA-3’
Mouse *Tgfb1*	Reversed	5’-CGTGGAGTTTGTTATCTTTGCTG-3’
Mouse *Cdh1*	Forward	5’-AGAGAAGCCATTGCCAAGTAC-3’
Mouse *Cdh1*	Reversed	5’-AACGAATCCCTCAAAGACCG-3’

Mouse *Has2*	Forward	5'-AGTCATGTACACAGCCTTCAG-3'
Mouse *Has2*	Reversed	5'-CTCCAACACCTCCAACCATAG-3'
Mouse *Zdhhc1*	Forward	5'-CACAAGGAGCTGGAGTCATG-3'
Mouse *Zdhhc1*	Reversed	5'-TGGCAAGAAACTGGGAAGG-3'
Mouse *Zdhhc2*	Forward	5'-GCAGCTATGTTTTCTGTCAGC-3'
Mouse *Zdhhc2*	Reversed	5'-ATCCGTTCTTATCTGTTCCGTG-3'
Mouse *Zdhhc3*	Forward	5'-CCCTGCTCTTCCTCATTTTCAC-3'
Mouse *Zdhhc3*	Reversed	5'-CACGGCTTTCATGTTCATCC-3'
Mouse *Zdhhc4*	Forward	5'-GGAAACTTACCTAGATGACGTGG-3'
Mouse *Zdhhc4*	Reversed	5'-CAAAACGATGACAAAGCCCAG-3'
Mouse *Zdhhc5*	Forward	5'-GGTGCGAATGAAATGGTGTG-3'
Mouse *Zdhhc5*	Reversed	5'-GTATCTGTAGTTCCTGCGACC-3'
Mouse *Zdhhc6*	Forward	5'-AGGAGAGTTCATCTTAGCAACG-3'
Mouse *Zdhhc6*	Reversed	5'-ACCATCACAGGGACACTTTTC-3'
Mouse *Zdhhc7*	Forward	5'-CAAAGACTTCTGGTACTCCGTG-3'
Mouse *Zdhhc7*	Reversed	5'-CTCTCCATGTACTCCTTCGTG-3'
Mouse *Zdhhc8*	Forward	5'-CATCCTCTTCCTCTTTGTCCTG-3'
Mouse *Zdhhc8*	Reversed	5'-CCGCACATCCACATTCTTG-3'
Mouse *Zdhhc9*	Forward	5'-CCGCTACTTCTACCTCTTCATC-3'
Mouse *Zdhhc9*	Reversed	5'-GTACTTCCAGAACAGTTCCAGG-3'
Mouse *Zdhhc12*	Forward	5'-CTTAACTCTGGGATGCTGGTG-3'
Mouse *Zdhhc12*	Reversed	5'-GCTCTTCCCATTGGCGTAG-3'
Mouse *Zdhhc13*	Forward	5'-TCACATGCTAAGAACAGAGGC-3'
Mouse *Zdhhc13*	Reversed	5'-ATGTATCCAACAGCCCACAG-3'
Mouse *Zdhhc14*	Forward	5'-GGATTCCTCGATGCCCTTAAG-3'
Mouse *Zdhhc14*	Reversed	5'-TGTCTTCGTTTGTGGTCTGG-3'
Mouse *Zdhhc15*	Forward	5'-CGCTCTAAGTTCCATGTCCTC-3'
Mouse *Zdhhc15*	Reversed	5'-TGGTCCACTTGTGAATACTGG-3'
Mouse *Zdhhc16*	Forward	5'-GGGCAGGGTGTTTAGGAATC-3'
Mouse *Zdhhc16*	Reversed	5'-GGGCAGGTGATTAGAAGGTAAC-3'
Mouse *Zdhhc17*	Forward	5'-GATGAGTACGAGACCGAAACG-3'
Mouse *Zdhhc17*	Reversed	5'-GACGATGTCCCATGTGCTATAG-3'
Mouse *Zdhhc19*	Forward	5'-TGTCACTCTGTCTCTACTCGG-3'
Mouse *Zdhhc19*	Reversed	5'-GCAGGAAGAGCGGAATCAAA-3'
Mouse *Zdhhc20*	Forward	5'-GAAGGAGCGTTATGAGAAGGAG-3'
Mouse *Zdhhc20*	Reversed	5'-GGGCTCGGTCAGGTTTAATC-3'
Mouse *Zdhhc21*	Forward	5'-CCCAAAATTGTCCTCTTTCCTC-3'
Mouse *Zdhhc21*	Reversed	5'-TTGGGATTTTCAGGGAGTCTTC-3'
Mouse *Zdhhc23*	Forward	5'-GATATGCGGTATCTGTGTACGG-3'
Mouse *Zdhhc23*	Reversed	5'-TTCAAGGTCAGCGATATTCCG-3'
Mouse *Zdhhc24*	Forward	5'-TCTACACAGTGGCTCTCCTG-3'
Mouse *Zdhhc24*	Reversed	5'-GCATCCCATGGAAAAGCAG-3'
Mouse *Ccl1*	Forward	5'-CTTCCCCTGAAGTTTATCCAGT-3'
Mouse *Ccl1*	Reversed	5'-TCTACCTTTGTTCAGCCTGAAT-3'

Mouse *Ccl2*	Forward	5’-TTTTTGTCACCAAGCTCAAGAG-3’
Mouse *Ccl2*	Reversed	5’-TTCTGATCTCATTTGGTTCCGA-3’
Mouse *Ccl3*	Forward	5’-TTGCTGTTCTTCTCTGTACCAT-3’
Mouse *Ccl3*	Reversed	5’-AATAGTCAACGATGAATTGGCG-3’
Mouse *Ccl4*	Forward	5’-ACTTCCTGCTGTTTCTCTTACA-3’
Mouse *Ccl4*	Reversed	5’-CCAAGTCACTCATGTACTCAGT-3’
Mouse *Ccl5*	Forward	5’-GTATTTCTACACCAGCAGCAAG-3’
Mouse *Ccl5*	Reversed	5’-TCTTGAACCCACTTCTTCTCTG-3’
Mouse *Ilβ*	Forward	5’-ATCTCGCAGCAGCACATCAA-3’
Mouse *Ilβ*	Reversed	5’-ATGGGAACGTCACACACCAG-3’
Mouse *Il6*	Forward	5’-CTCCCAACAGACCTGTCTATAC-3’
Mouse *Il6*	Reversed	5’-CCATTGCACAACTCTTTTCTCA-3’
Mouse *Il18*	Forward	5’-AGACCTGGAATCAGACAACTTT-3’
Mouse *Il18*	Reversed	5’-TCAGTCATATCCTCGAACACAG-3’
Mouse *Tnfα*	Forward	5’-ATGTCTCAGCCTCTTCTCATTC-3’
Mouse *Tnfα*	Reversed	5’-GCTTGTCACTCGAATTTTGAGA-3’
Mouse *Gapdh*	Forward	5’-CCCTTATTGACCTCAACTACATGGT-3’
Mouse *Gapdh*	Reversed	5’-GAGGGGCCATCCACAGTCTTCTG-3’

Supplemental Table 5. List of ChIP-PCR primer

Name	Forward	Reverse
*Snai1*_ enhancer	agactggaataccctcctctcc	ttctcaaaggggctgtcacc
*Has2*_ enhancer	ctgcatccctgagtcattgt	aggtctgccttgagttgtaag
