# Supplementary material for: ZDHHC18 promotes renal fibrosis development by regulating HRAS palmitoylation
Source: J Clin Invest. 2025 Feb 4;135(6):e180242. doi: 10.1172/JCI180242 (PMC11910235; doi:10.1172/JCI180242)

Unedited blot and gel images

Fig.2C

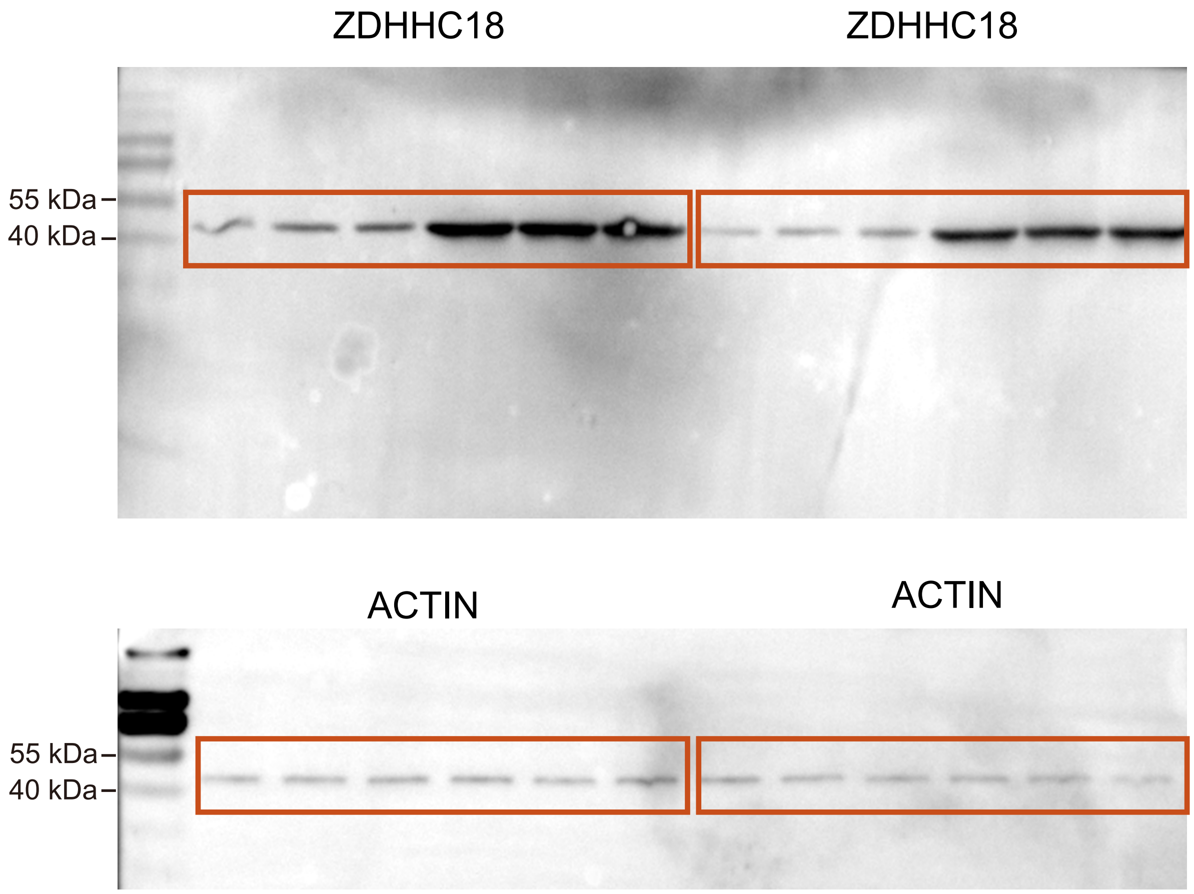

Fig.6B

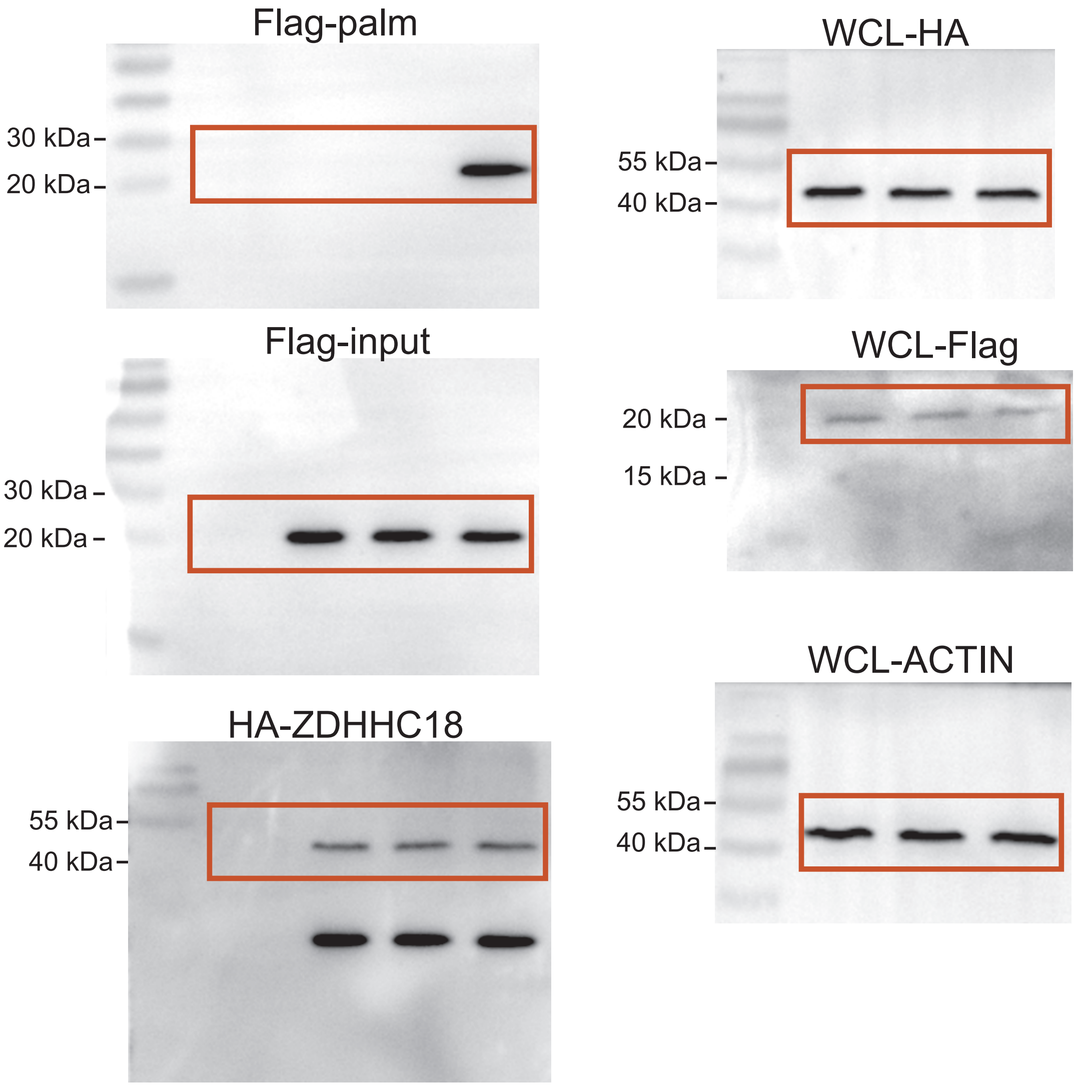
Flag-palm
30 kDa
20 kDa
WCL-HA
55 kDa
40 kDa
Flag-input
30 kDa
20 kDa
WCL-Flag
20 kDa
15 kDa
WCL-ACTIN
55 kDa
40 kDa
HA-ZDHHC18
55 kDa
40 kDa

Fig.6C

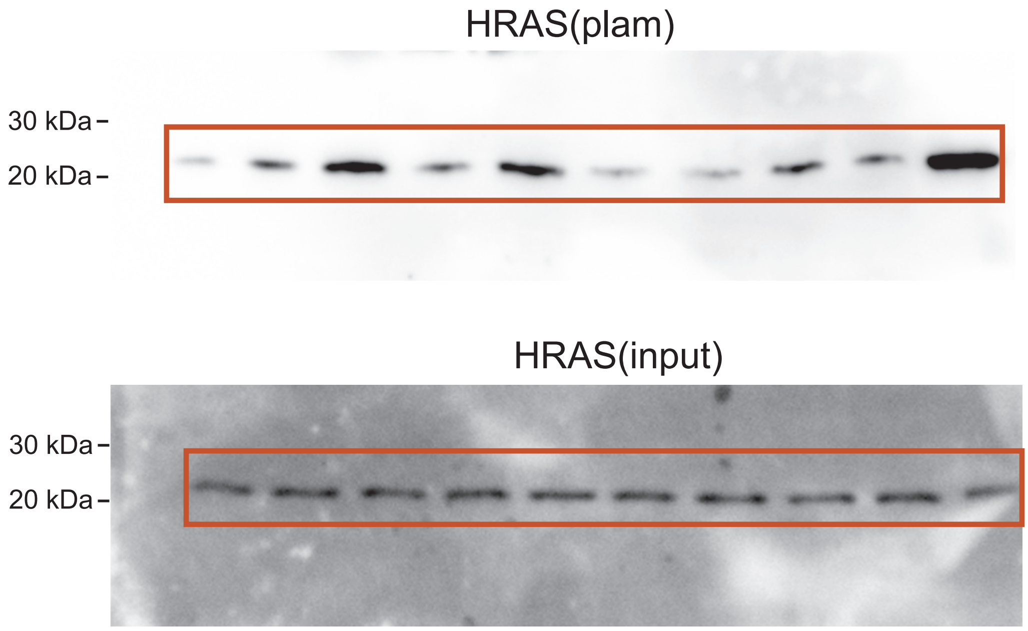

Fig.6D

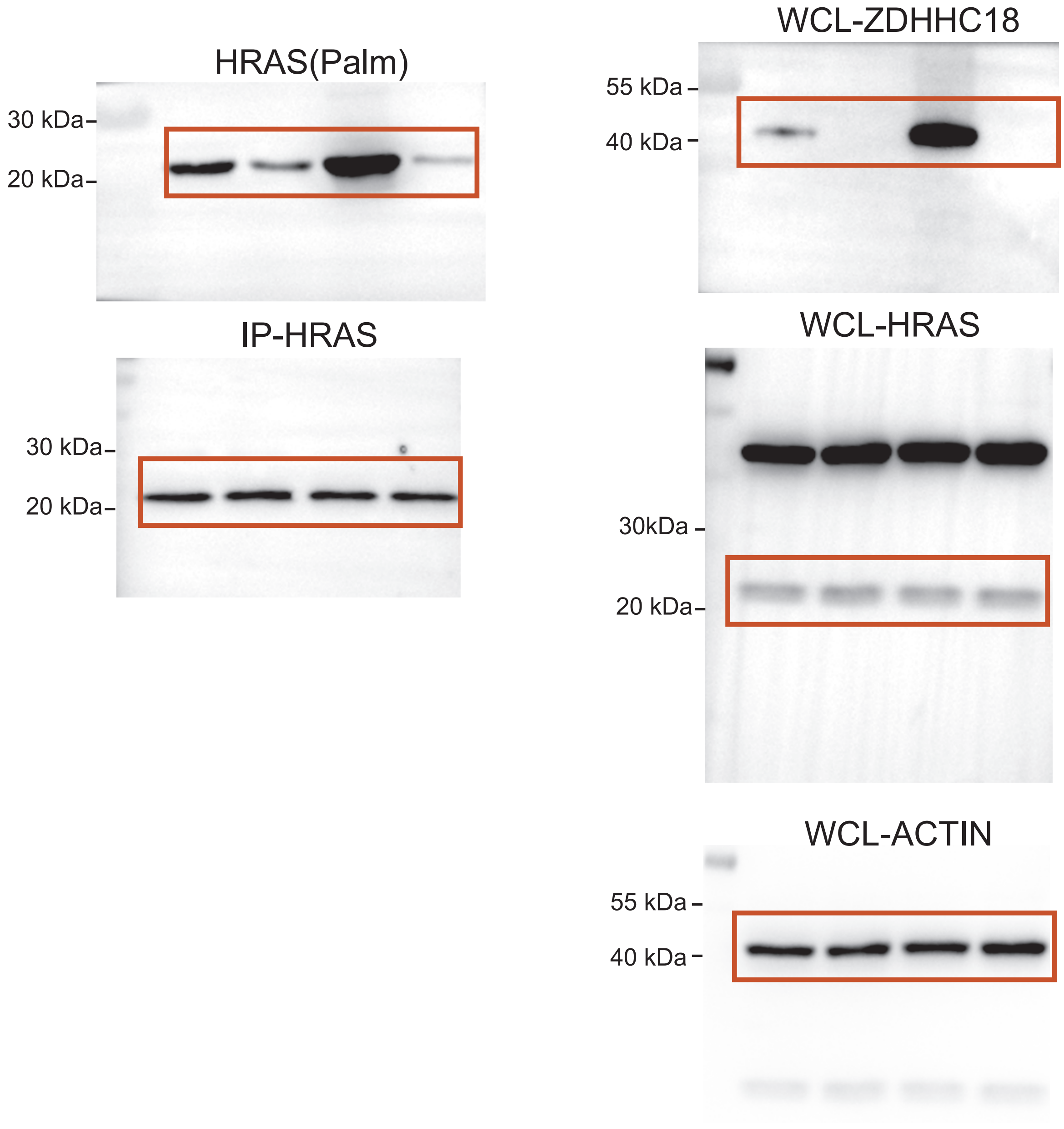
HRAS(Palm)
30 kDa
20 kDa
IP-HRAS
30 kDa
20 kDa
WCL-ZDHHC18
55 kDa
40 kDa
WCL-HRAS
30kDa
20 kDa
WCL-ACTIN
55 kDa
40 kDa

Fig.6E

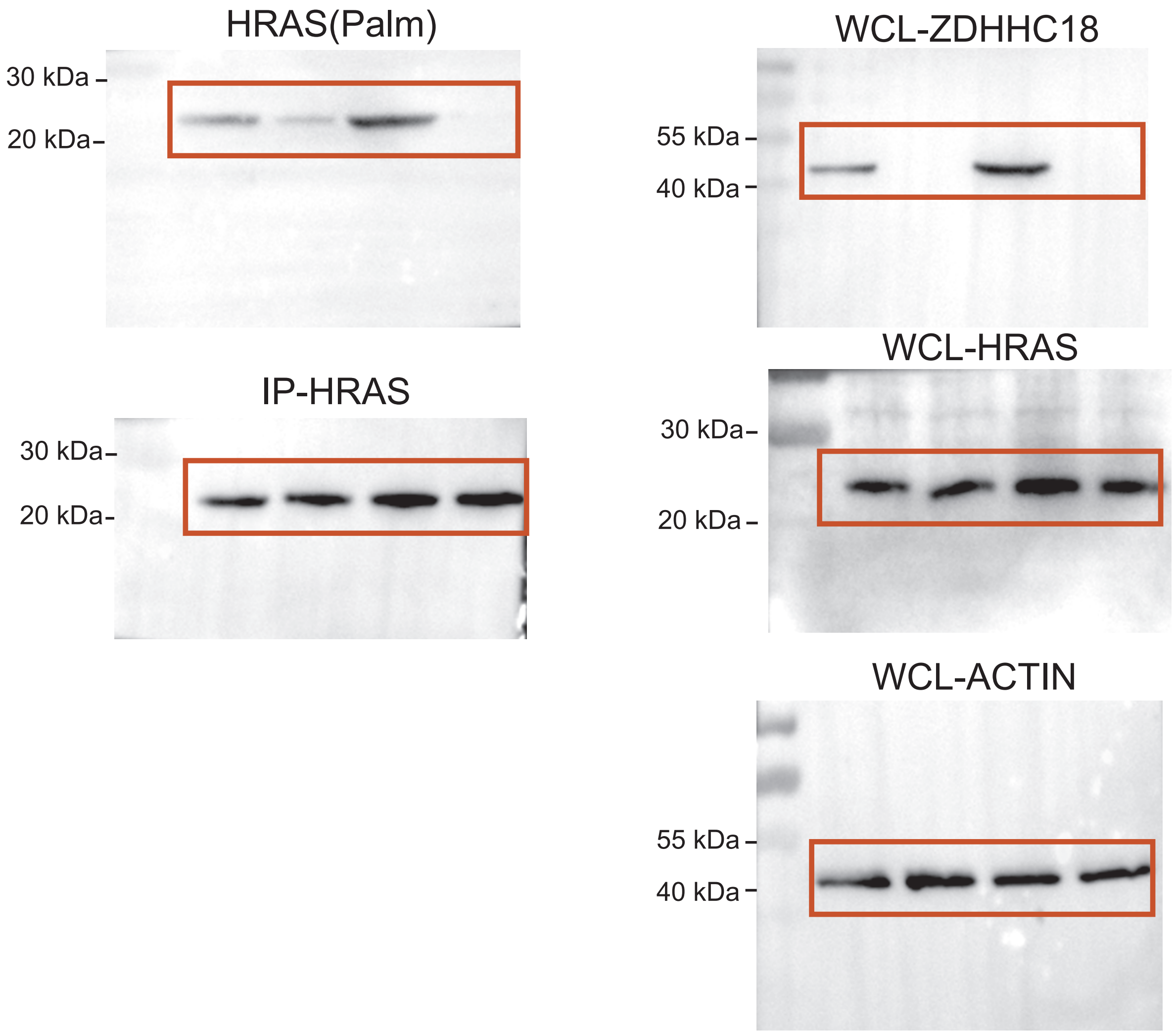

HRAS(Palm)
30 kDa
20 kDa
IP-HRAS
30 kDa
20 kDa
WCL-ZDHHC18
55 kDa
40 kDa
WCL-HRAS
30 kDa
20 kDa
WCL-ACTIN
55 kDa
40 kDa

Fig.6F

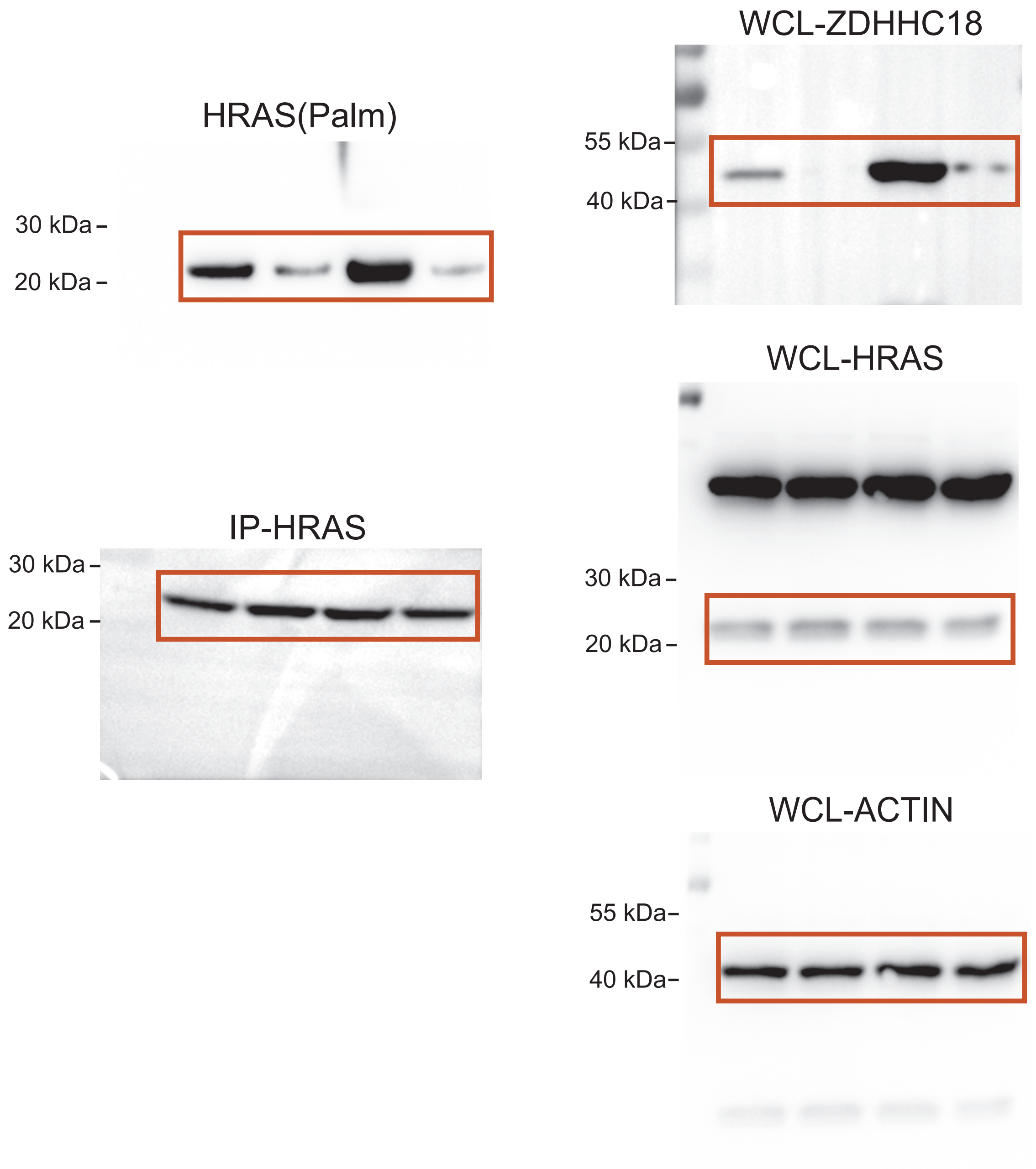
HRAS(Palm)
30 kDa
20 kDa
IP-HRAS
30 kDa
20 kDa
WCL-ZDHHC18
55 kDa
40 kDa
WCL-HRAS
30 kDa
20 kDa
WCL-ACTIN
55 kDa
40 kDa

Fig.6G

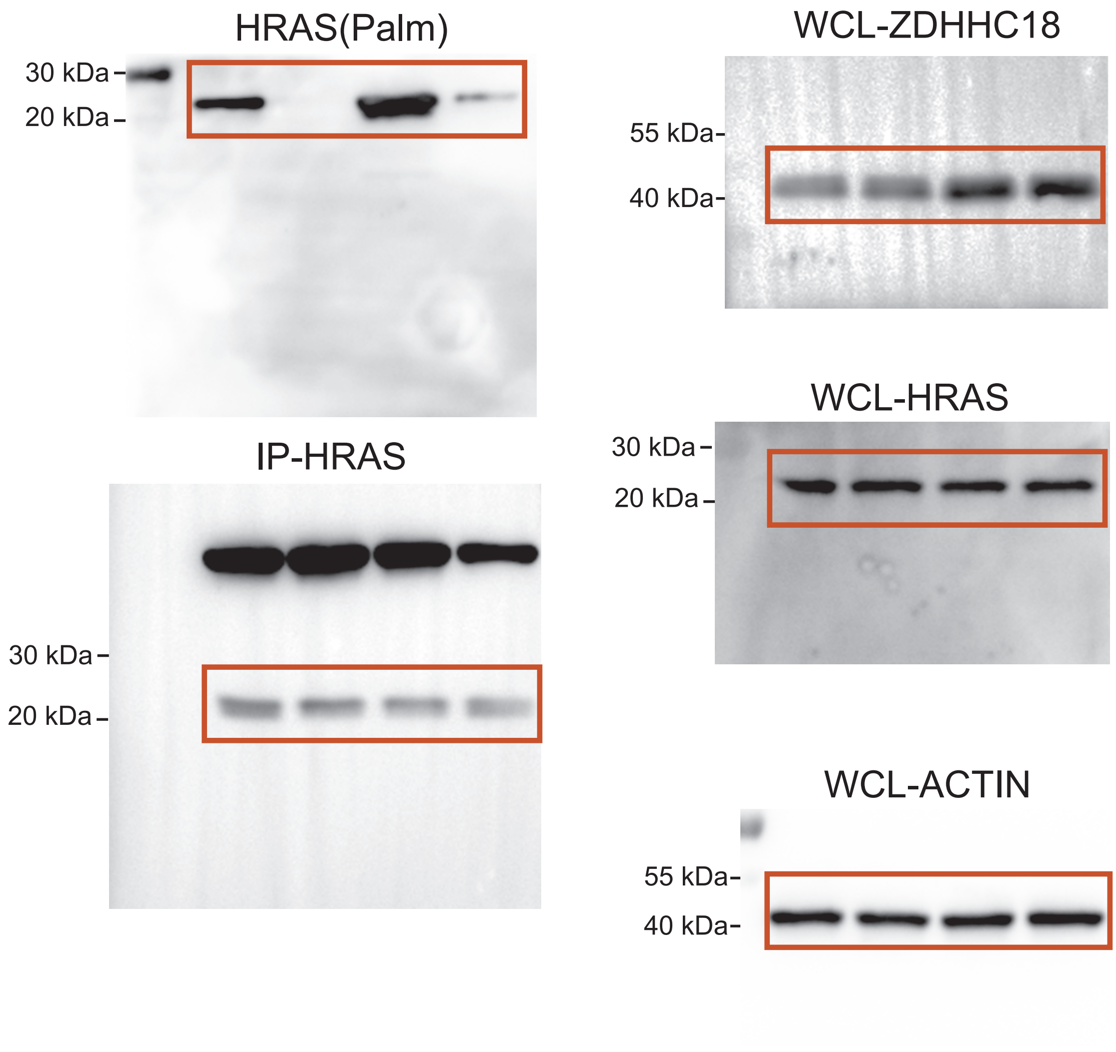
HRAS(Palm)
30 kDa
20 kDa
IP-HRAS
30 kDa
20 kDa
WCL-ZDHHC18
55 kDa
40 kDa
WCL-HRAS
30 kDa
20 kDa
WCL-ACTIN
55 kDa
40 kDa

Fig.6I

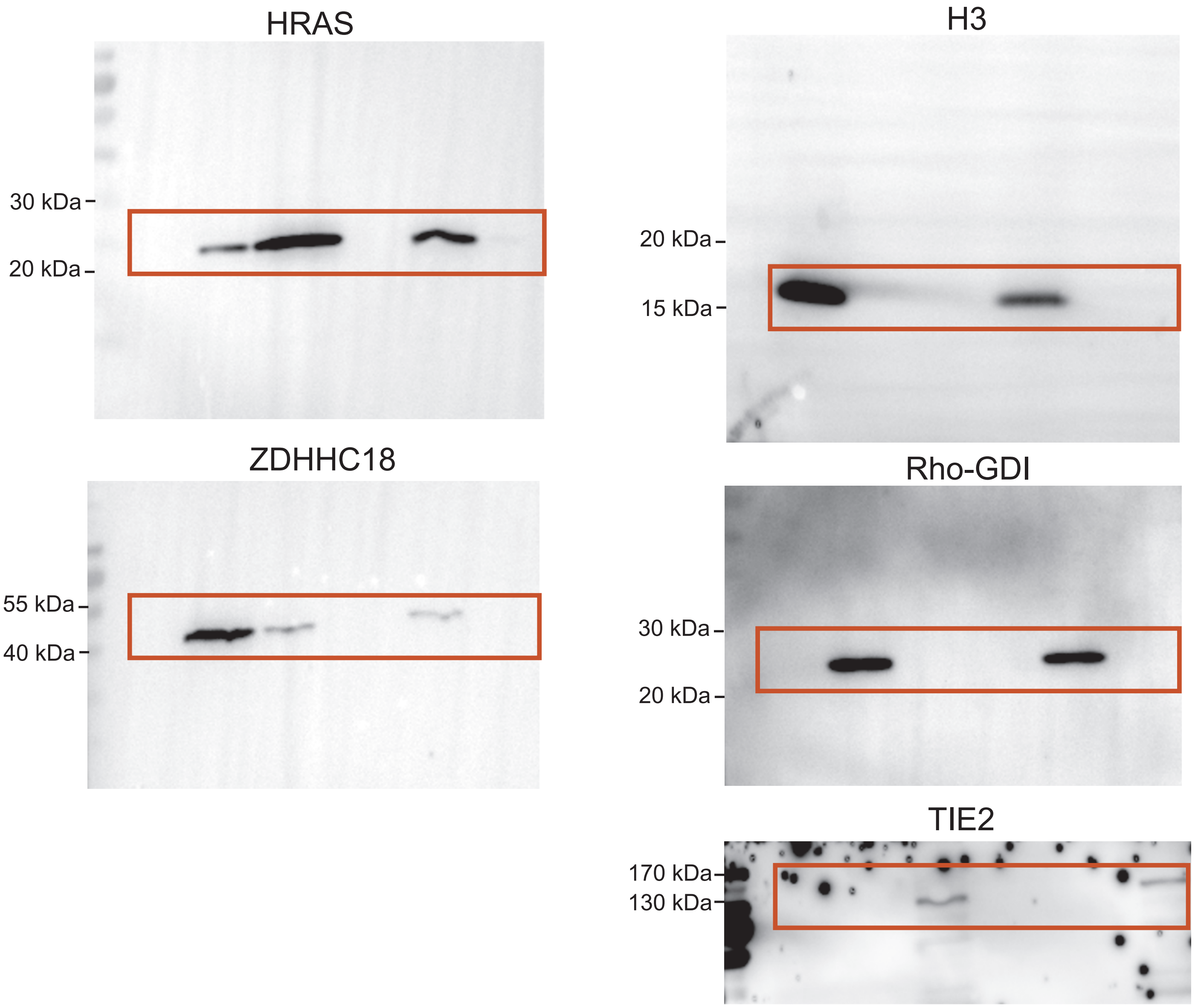
HRAS
30 kDa
20 kDa
H3
20 kDa
15 kDa
ZDHHC18
55 kDa
40 kDa
Rho-GDI
30 kDa
20 kDa
TIE2
170 kDa
130 kDa

Fig.7A

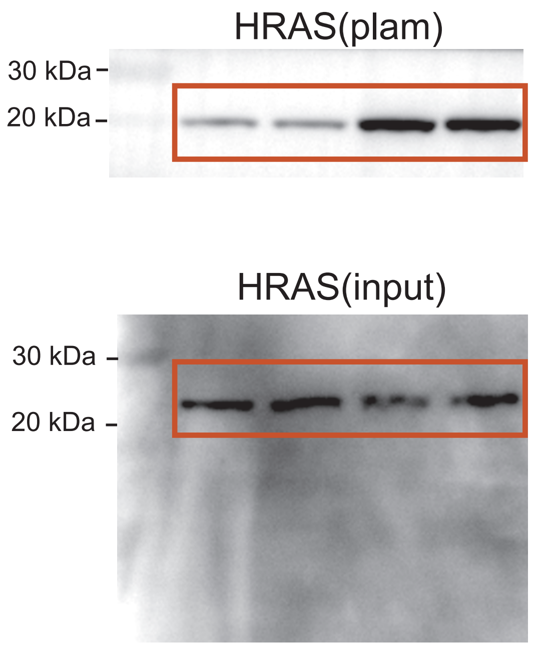

Fig.7B

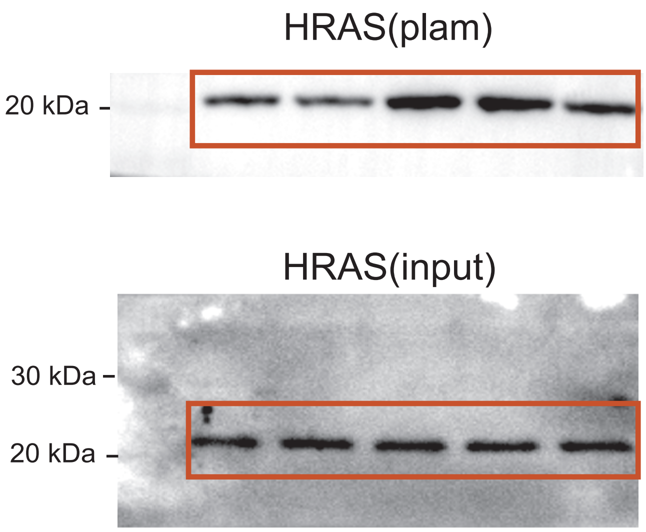

Fig.7C

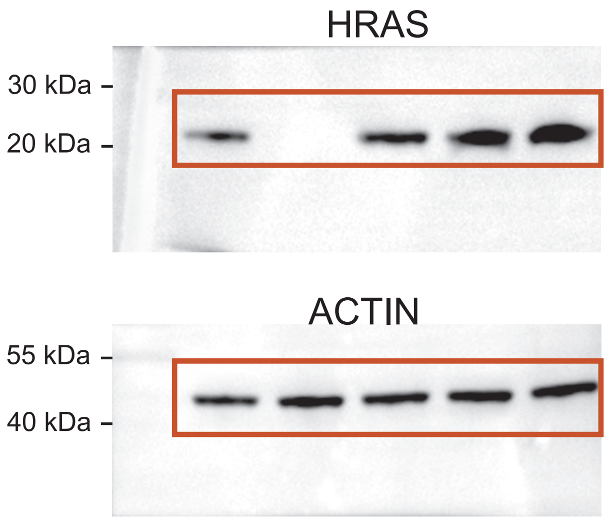

Fig.7E

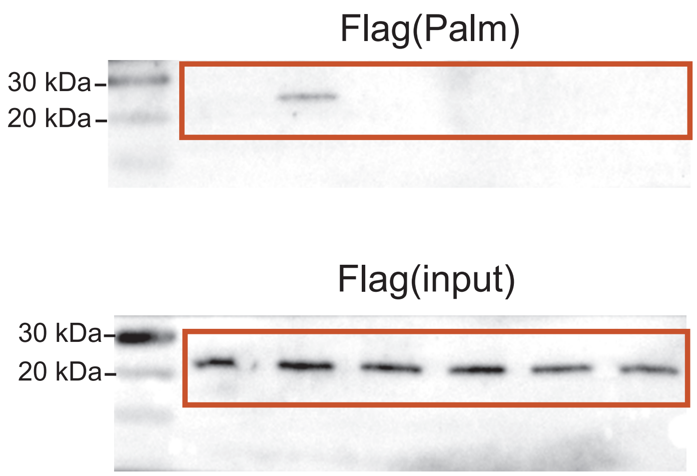

Full unedited blot/gel for Figure 7H

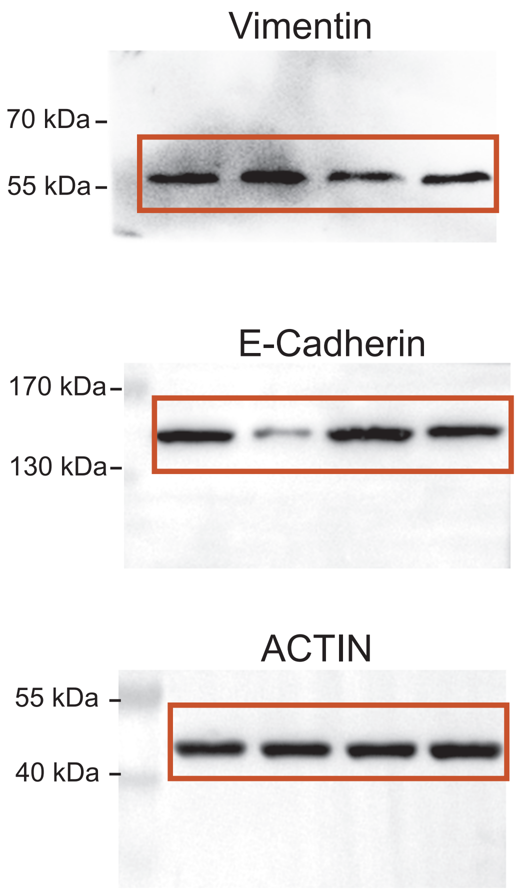

Fig.8A

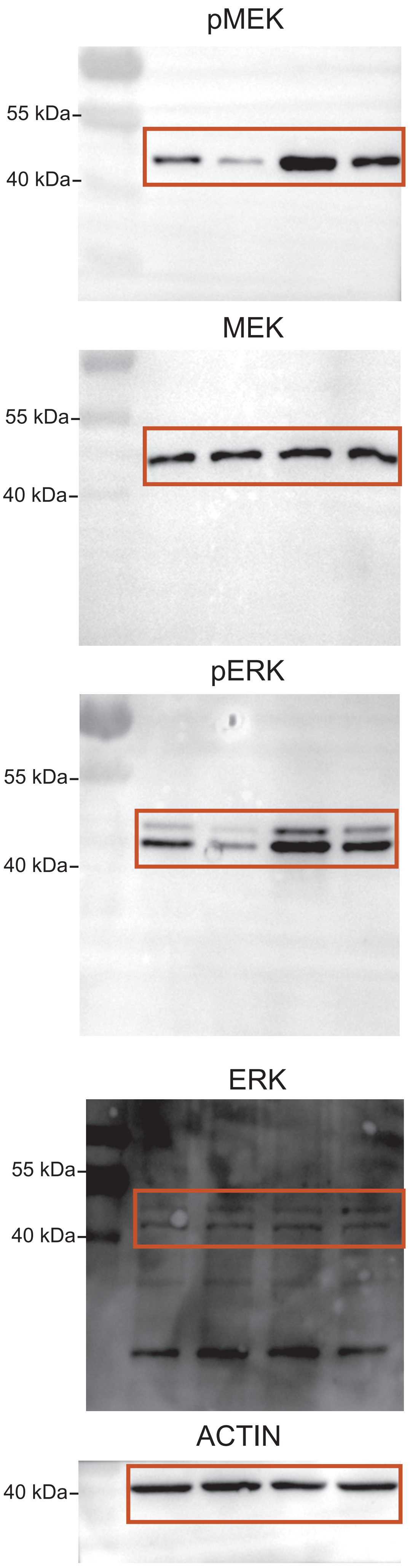
pMEK
55 kDa
40 kDa
MEK
55 kDa
40 kDa
pERK
55 kDa
40 kDa
ERK
55 kDa
40 kDa
ACTIN
40 kDa

Fig.8C

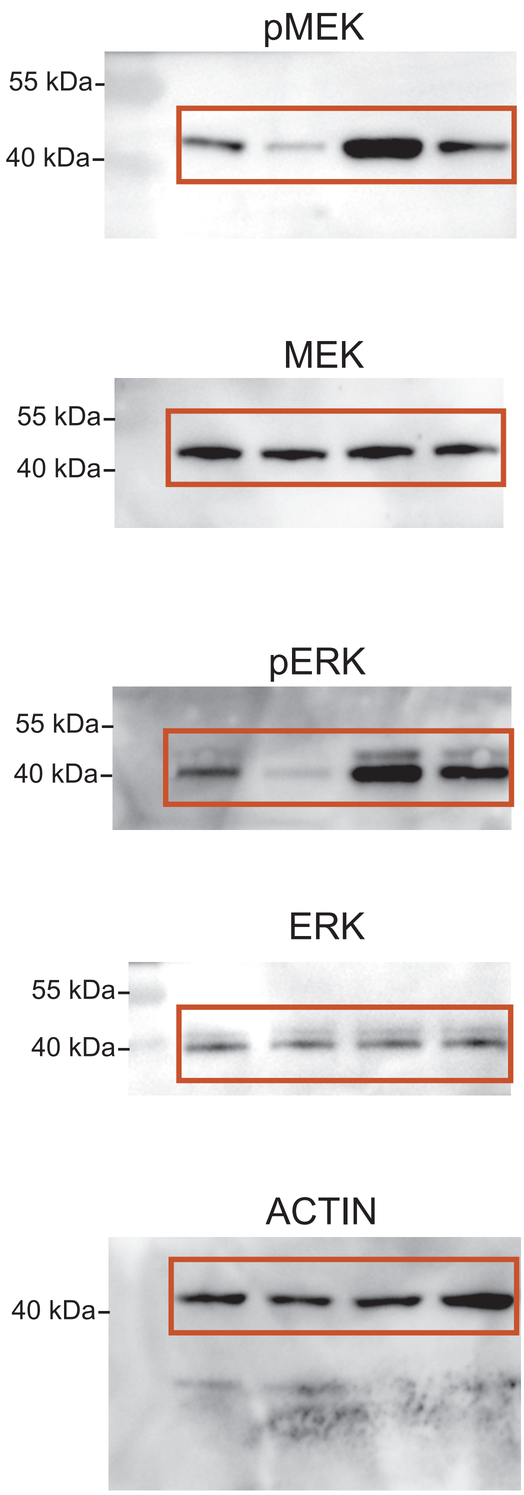
pMEK
55 kDa
40 kDa
MEK
55 kDa
40 kDa
pERK
55 kDa
40 kDa
ERK
55 kDa
40 kDa
ACTIN
40 kDa

Fig.8G

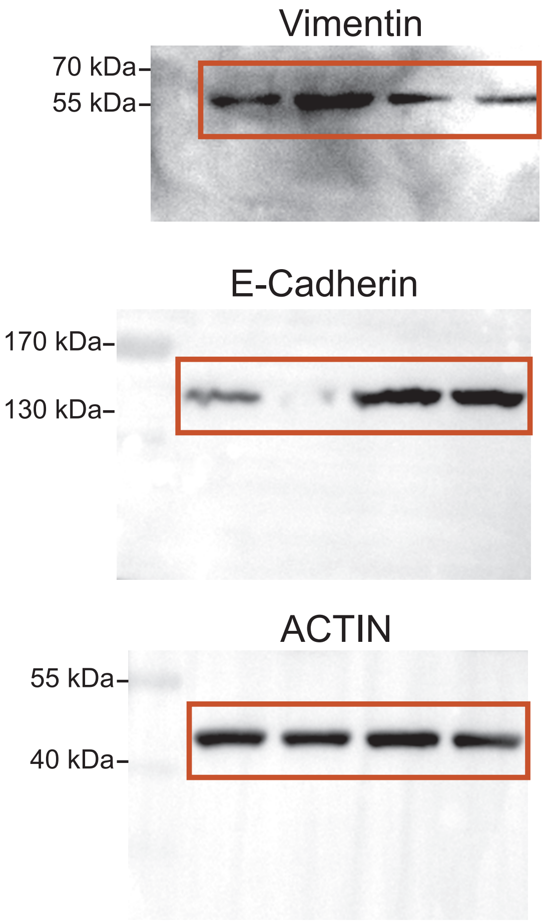
Vimentin
70 kDa
55 kDa
E-Cadherin
170 kDa
130 kDa
ACTIN
55 kDa
40 kDa

Fig.8H

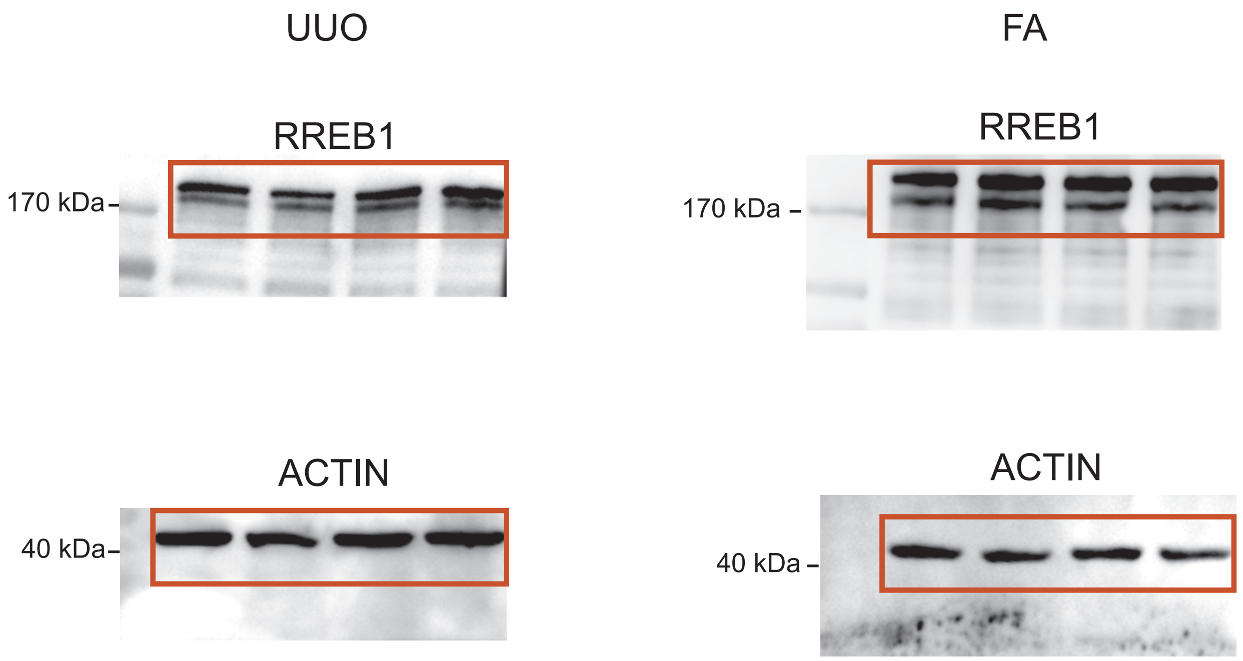

Fig.8K

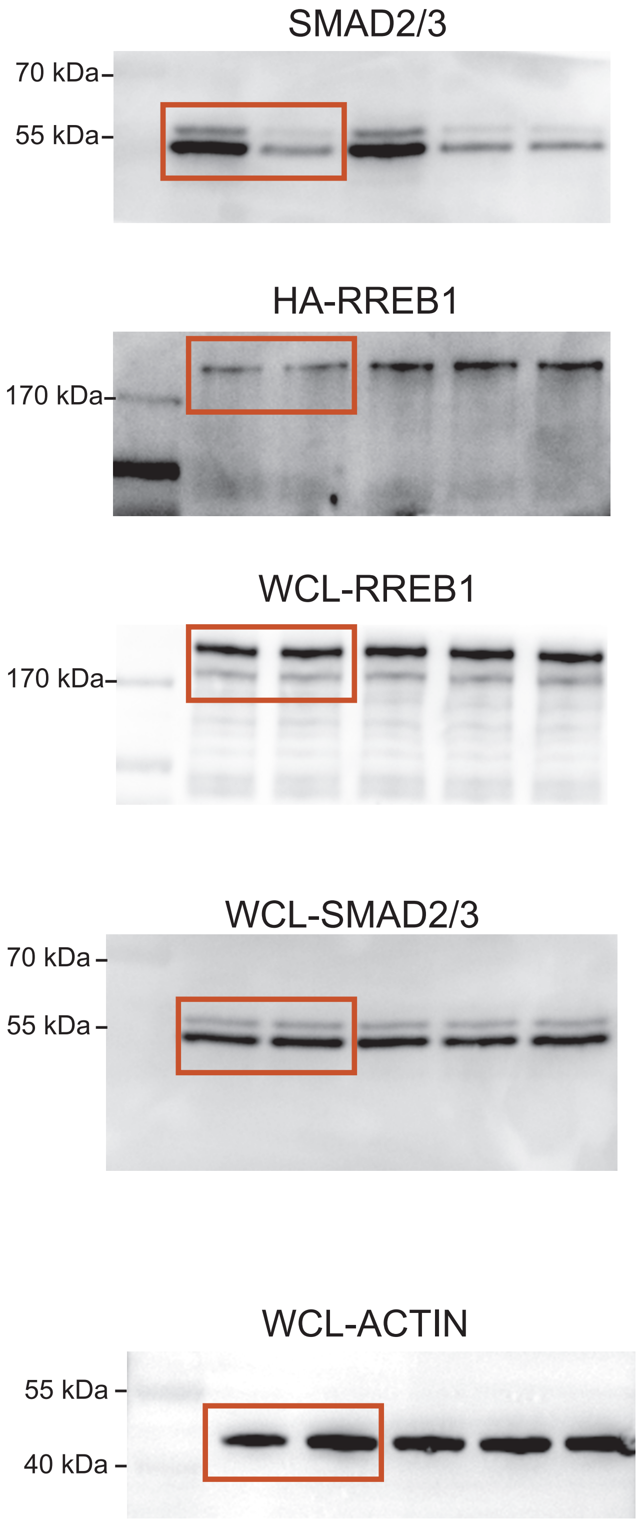
SMAD2/3
70 kDa
55 kDa
HA-RREB1
170 kDa
WCL-RREB1
170 kDa
WCL-SMAD2/3
70 kDa
55 kDa
WCL-ACTIN
55 kDa
40 kDa

Fig.8P

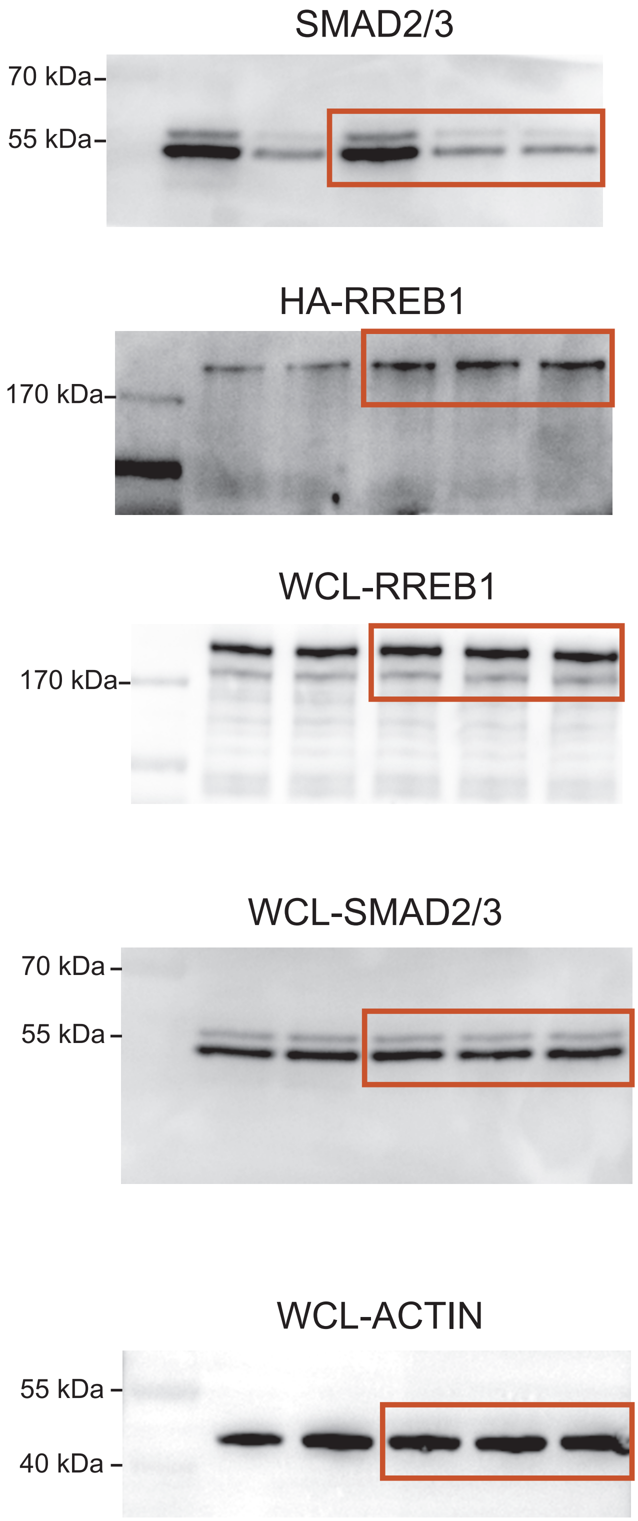
SMAD2/3
70 kDa
55 kDa
HA-RREB1
170 kDa
WCL-RREB1
170 kDa
WCL-SMAD2/3
70 kDa
55 kDa
WCL-ACTIN
55 kDa
40 kDa

Fig.S3B

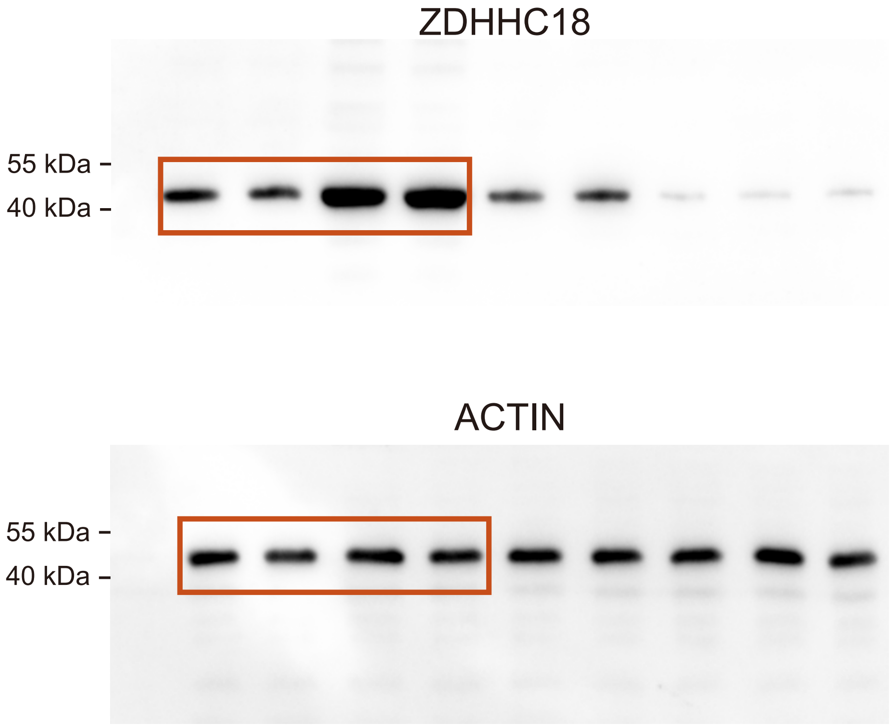

Fig.S3D

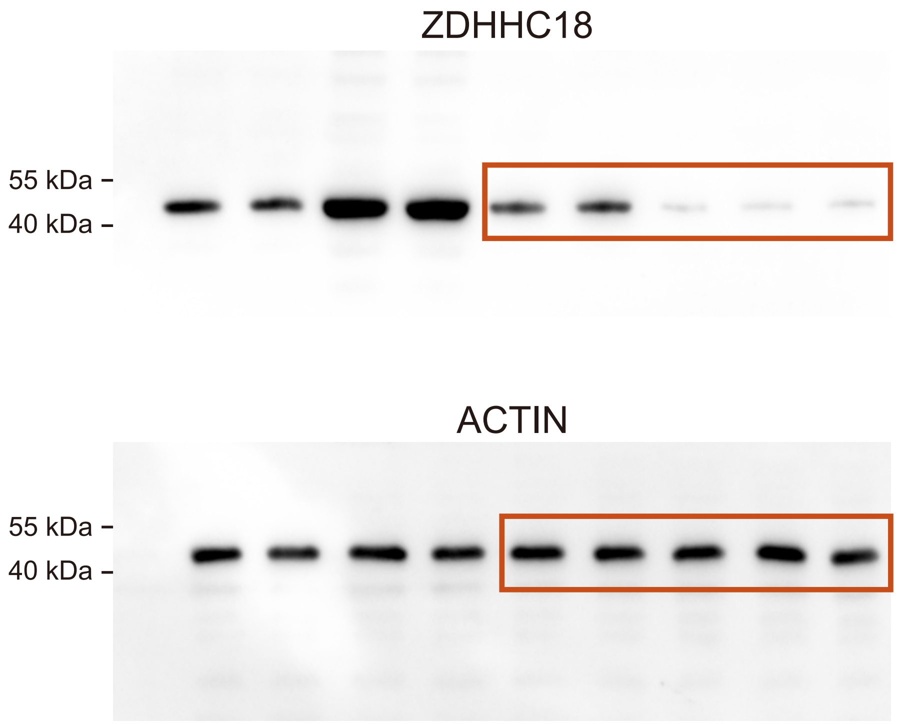

Fig.S3H

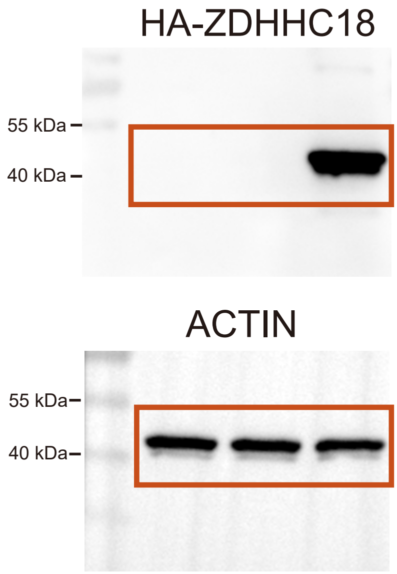

Fig.S4C

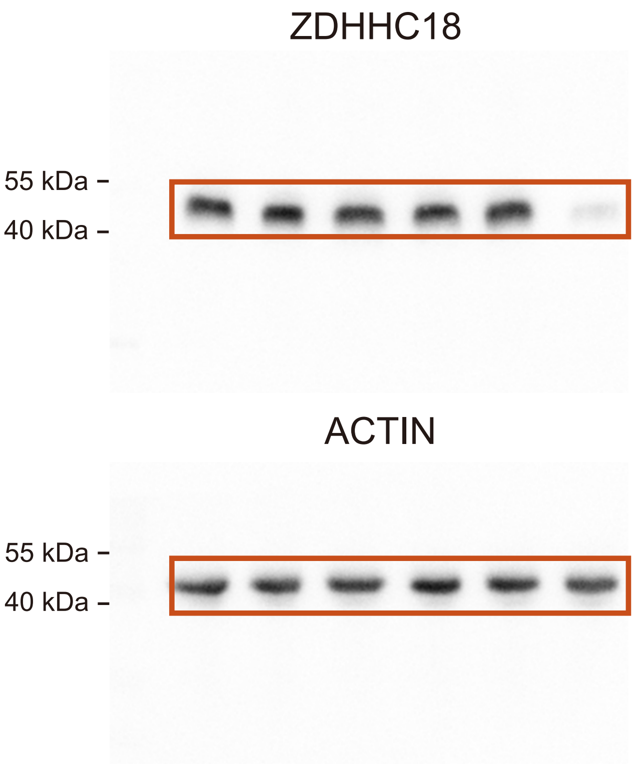

Supplement: Unedited blot and gel images [file jci-135-180242-s015.pdf]
